# Characteristics of headaches attributed to SARS-CoV-2 vaccination and factors associated with its frequency and prolongation: a cross-sectional cohort study

**DOI:** 10.3389/fneur.2023.1214501

**Published:** 2023-08-02

**Authors:** Melika Jameie, Mansoureh Togha, Mehdi Azizmohammad Looha, Elham Jafari, Mohammad Yazdan Panah, Nima Hemmati, Somayeh Nasergivehchi

**Affiliations:** ^1^Iranian Center of Neurological Research, Neuroscience Institute, Tehran University of Medical Sciences, Tehran, Iran; ^2^Neuroscience Research Center, Iran University of Medical Sciences, Tehran, Iran; ^3^Department of Headache, Iranian Center of Neurological Research, Neuroscience Institute, Tehran University of Medical Sciences, Tehran, Iran; ^4^Neurology Ward, Sina Hospital, School of Medicine, Tehran University of Medical Sciences, Tehran, Iran; ^5^Basic and Molecular Epidemiology of Gastrointestinal Disorders Research Center, Research Institute for Gastroenterology and Liver Diseases, Shahid Beheshti University of Medical Sciences, Tehran, Iran; ^6^School of Medicine, Shahrekord University of Medical Sciences, Shahrekord, Iran; ^7^Minimally Invasive Surgery Research Centre, Iran University of Medical Sciences, Tehran, Iran

**Keywords:** headache, headache disorders, COVID-19 vaccines, COVID-19, SARS-CoV-2, vaccination, adverse event, safety

## Abstract

**Background:**

Headache is the most frequent neurological adverse event following SARS-CoV-2 vaccines. We investigated the frequency, characteristics, and factors associated with post-vaccination headaches, including their occurrence and prolongation (≥ 48 h).

**Methods:**

In this observational cross-sectional cohort study, retrospective data collected between April 2021–March 2022 were analyzed. Univariate and multivariate logistic regressions were used to evaluate the effect of clinicodemographic factors on the odds of post-vaccination headache occurrence and prolongation.

**Results:**

Of 2,500 people who were randomly sent the questionnaire, 1822 (mean age: 34.49 ± 11.09, female: 71.5%) were included. Headache prevalence following the first (V_1_), second (V_2_), and third (V_3_) dose was 36.5, 23.3, and 21.7%, respectively (*p* < 0.001). Post-vaccination headaches were mainly tension-type (46.5%), followed by migraine-like (36.1%). Headaches were mainly bilateral (69.7%), pressing (54.3%), moderate (51.0%), and analgesic-responsive (63.0%). They mainly initiated 10 h [4.0, 24.0] after vaccination and lasted 24 h [4.0, 48.0]. After adjusting for age and sex, primary headaches (V_1_: aOR: 1.32 [95%CI: 1.08, 1.62], V_2_: 1.64 [1.15, 2.35]), post-COVID-19 headaches (V_2_: 2.02 [1.26, 3.31], V_3_: 2.83 [1.17, 7.47]), headaches following the previous dose (V_1_ for V_2_: 30.52 [19.29, 50.15], V_1_ for V_3_: 3.78 [1.80, 7.96], V_2_ for V_3_: 12.41 [4.73, 35.88]), vector vaccines (V_1_: 3.88 [3.07, 4.92], V_2_: 2.44 [1.70, 3.52], V_3_: 4.34 [1.78, 12.29]), and post-vaccination fever (V_1_: 4.72 [3.79, 5.90], V_2_: 6.85 [4.68, 10.10], V_3_: 9.74 [4.56, 22.10]) increased the odds of post-vaccination headaches. Furthermore, while primary headaches (V_1_: 0.63 [0.44, 0.90]) and post-COVID-19 headaches (V_1_: 0.01 [0.00, 0.05]) reduced the odds of prolonged post-vaccination headaches, psychiatric disorders (V_1_: 2.58 [1.05, 6.45]), headaches lasting ≥48 h following the previous dose (V_1_ for V_2_: 3.10 [1.08, 10.31]), and migraine-like headaches at the same dose (V_3_: 5.39 [1.15, 32.47]) increased this odds.

**Conclusion:**

Patients with primary headaches, post-COVID-19 headaches, or headaches following the previous dose, as well as vector-vaccine receivers and those with post-vaccination fever, were at increased risk of post-SARS-CoV-2-vaccination headaches. Primary headaches and post-COVID-19 headaches reduced the odds of prolonged post-vaccination headaches. However, longer-lasting headaches following the previous dose, migraine-like headaches at the same dose, and psychiatric disorders increased this odd.

## 1. Introduction

Headache disorders are among the most prevalent and debilitating conditions worldwide, accounting for 1.82 and 5.37% of total disability-adjusted life years (DALYs) and years lost to disability (YLDs), respectively (1–3). Globally, the prevalence of active headache disorders among adults is estimated to be over 50% (4, 5). Since the advent of coronavirus disease-2019 (COVID-19), caused by the severe acute respiratory syndrome coronavirus 2 (SARS-CoV-2), an array of associated neurological symptoms, including headaches, have been documented (6–10). Approved and authorized SARS-COV-2 vaccines are the most effective and safest tools for preventing COVID-19-related morbidity and mortality (11). As of January 2023, more than 13 billion SARS-CoV-2 vaccine doses have been administered (12). Nevertheless, concerns over the neurological adverse events (AEs) following SARS-CoV-2 vaccination were disclosed, of which headaches were the most frequent (13–15).

With an estimated incidence rate of 93.696 per 100,000 per year, headaches are the most frequent neurological manifestation following SARS-CoV-2 vaccination (16), affecting approximately 20–40% of individuals (17–20), and even higher in those with a previous history of headache disorders (21–23). According to a recent meta-analysis, SARS-CoV-2 vaccines were associated with a two-fold increased risk of headache within seven days of administration (19). Headaches are also reported as common AEs following other vaccines, including influenza, bivalent meningococcal group B vaccine, quadrivalent meningococcal diphtheria toxoid conjugate vaccine, and quadrivalent human papillomavirus vaccine (20, 24). Nevertheless, according to the international classification of headache disorders (ICHD-3), no classification or diagnostic features have been specifically defined for vaccine-related headaches so far (25).

Understanding the characteristics of post-vaccination headaches is of immense importance since while headaches are usually considered non-serious, they can also be a sign of life-threatening conditions, including cerebral venous thrombosis (CVT) (26), acute myelitis (27), and intracerebral hemorrhage (28). Although vaccine-related headaches are frequently reported, there are few studies thoroughly describing the characteristics of headaches following the SARS-COV-2 vaccination (19, 23, 29–32). Moreover, while factors associated with post-COVID-19 infection headaches have been widely investigated (33–35), to our knowledge, factors associated with post-SARS-CoV-2 vaccination headaches occurrence (20, 29, 35) and particularly *prolongation* are much less discussed in the literature, and there are still conflicting findings in this area.

In light of this information, we aimed to investigate the frequency and characteristics of headaches attributed to SARS-CoV-2 vaccination, determine factors associated with developing headaches following SARS-CoV-2 vaccination, and identify the factors related to prolonged headaches (≥ 48 h) among SARS-CoV-2 vaccine receivers. In particular, we sought to investigate which characteristics of the patient’s previous headaches (primary headaches, headaches after COVID-19, and headaches after the previous vaccine doses) could predict the occurrence and prolongation of headaches after receiving the following the 1st (V_1_), 2nd (V_2_), and 3rd (V_3_) dose of SARS-CoV-2 vaccines.

## 2. Materials and methods

### 2.1. Study design

In this Institutional Review Board-approved web-based, population-based cross-sectional cohort study, retrospective data (April 2021–March 2022) were analyzed. Patients gave their informed consent for participation and publishing, according to the Declaration of Helsinki (36). The individuals who were solicited to participate in our study were randomly selected from a pool of patients within the healthcare system who had documented vaccine immunization. From this pool, 2,500 individuals who had received at least one vaccine dose (1st or 2nd or 3rd) in the last month were randomly chosen. The reason for choosing a one-month interval was to ensure that the time between answering the survey and receiving the vaccine was neither too short, as headaches could develop *after* answering the survey or persist *beyond* the response date, nor too long, as it could increase recall bias. Additionally, in case of long intervals, respondents may associate headaches attributed to other causes with the vaccine. An anonymous survey was distributed to the targeted vaccinated individuals, using a web-based link compatible with smartphones, tablets, laptops, and desktop PCs. Individuals were invited to volunteer for the survey using text messages and free social media platforms. Using social media platforms during the pandemic was a convenient way to increase participation in research projects (37). The purpose of the survey and the length of time it would take were explained to all invitees. The survey could be submitted after filling out the mandatory questions and filled out only once *via* the same device. The Alpha and Delta variants were the dominant SARS-CoV-2 variants at the time of this study, during which headache was one of the most common symptoms (38–40). This study accords with the Strengthening the Reporting of Observational Studies in Epidemiology (STROBE) statement^1^ (Supplementary Table S1).

### 2.2. Study population

Individuals aged ≥18 who had received at least one dose of any SARS-COV-2 vaccine type, were literate enough to fill the questionnaire, and volunteered to fill out the survey were considered eligible to be included, regardless of developing post-vaccination headaches. The following individuals were excluded: (a) pediatrics, (b) people who had a history of receiving a vaccine other than the SARS-CoV-2 vaccine in the last 3 months, (c) individuals with a history of substance or alcohol abuse due to their possible association with headaches (41, 42), (d) individuals who were reluctant to participate or did not provide informed consent, (e) those who answered the questions incompletely (responses missing ≥50% (43)), and (f) patients with immunocompromised conditions such as malignancies, solid organ transplantation, or inflammatory rheumatic diseases, as studies suggested different AE profile compared to immunocompetent patients (44).

### 2.3. Study objectives

There are three objectives of this study:

1. To investigate headache frequency and characteristics attributed to SARS-CoV-2 vaccination.
2. To determine factors associated with developing headaches following the 1st, 2nd, and 3rd doses of SARS-CoV-2 vaccination.
3. To determine factors associated with developing prolonged headaches (defined as headaches ≥48 h) following the 1st, 2nd, and 3rd dose of SARS-CoV-2 vaccination.

### 2.4. Study measures and definition of terms

The following information was provided, using a standardized checklist: (a) baseline demographic characteristics, (b) history and type of primary headache disorders, (c) COVID-19-related variables, (d) COVID-19-related headaches characteristics, (e) vaccine-related variables, and (f) variables attributed to the post-vaccination headaches (time to onset after SARS-CoV-2 vaccination, duration, intensity, day of experiencing the most severe headache, quality, localization, and lateralization, migraine-like accompanying symptoms, medications used, and resemblance to the either of the primary headache, post-COVID-19 headache, and headaches attributed to the previous vaccine doses).

#### 2.4.1. Headache-related definitions

Below we have provided the definitions of the used terms, as per the ICHD-3 guideline (Definition of Terms – ICHD-3).

*Primary headaches*: a headache disorder, not resulting from or attributed to another condition.

*Headaches attributed to COVID-19 infection*: “9. Headache attributed to infection – ICHD-3” is a subset of secondary headaches. The definitions and diagnostic criteria of this classification and its subdivisions, including “9.2 Headache attributed to systemic infection – ICHD-3” and “9.2.2 Headache attributed to systemic viral infection – ICHD-3” are provided in Supplementary Table S2.

*Headaches attributed to SARS-CoV-2 vaccination*: although post-vaccination headaches do not fully meet the diagnostic criteria for any specific category in the ICHD-3, the category of “8.1 Headache attributed to use of or exposure to a substance – ICHD-3” and its subcategories, including “8.1.9 Headache attributed to occasional use of non-headache medication – ICHD-3” and “8.1.11 Headache attributed to use of or exposure to other substance – ICHD-3” may bear some resemblance to vaccine-related headaches. The definitions and diagnostic criteria of these headaches are provided in Supplementary Table S3. These headaches initiate in a close temporal relationship (usually within minutes up to 12 h, according to the available literature) after exposure and usually resolve within 72 h. It is worth noting that the characteristics of these specific ICHD-3 subcategories (8.1.9 and 8.1.11) are still not well-defined in the existing literature, underscoring the need for further investigation into the characteristics of these headaches as well as headaches attributed to vaccination.

*Prolonged post-SARS-CoV-2 vaccination headache*: given the current absence of specific criteria for post-vaccination headaches and their duration, existing literature on headaches associated with SARS-CoV-2 vaccination suggests that they generally resolve within 24–36 h from the headache onset (19, 21, 29, 30, 45). On the other hand, post-vaccination headaches lasting more than 72 h are relatively uncommon (29). Furthermore, based on the ICHD-3 guideline, headaches attributed to the use of/exposure to substances typically resolve within 72 h. Thus, it appears that vaccine-related headaches typically resolve within 24 h and do not persist beyond 72 h. Hence, in this study, we classified headaches lasting 48 h or more as “prolonged” headaches.

*Resemblance of the patients’ post-vaccination headaches to their prior headaches*: subjectively assessed by a general inquiry, based on the patients’ own opinion, without specifically focusing on any particular characteristics.

*Headache type*: a standardized checklist according to the ICHD-3 was designed to classify the post-COVID-19 and post-SARS-CoV-2 vaccination headaches (25). Based on the patient’s answers to the questions, diagnoses of migraine-like and tension-type headaches (TTH) were performed (25). If the headache did not meet the criteria for specific headaches, it was categorized as undifferentiated.

*Headache intensity*: categorized based on the 11-point Numeric Rating Scale (NRS) scale as mild (NRS: 1–3, not very disturbing and no or little interfering with the daily work), moderate (NRS: 4–6, uncomfortable and significantly interfering with active daily living but lets the individual do daily work), and severe (NRS: 7–10, disabling or does not allow to perform daily work) (46). NRS is a validated and sensitive scale with a high test–retest reliability for measuring headache pain, where 0 corresponds to “no headache at all” and 10 to “the worst headache possible” (47).

*Headache quality*: reported as sharp pain, pressing, throbbing/pulsatile sensation, and dull ache.

*Headache location*: regions in the head, above the orbitomeatal line, and/or nuchal ridge affected by pain.

*Headache lateralization*: categorized as unilateral and bilateral. Unilateral headaches were defined as headaches affecting either the right or left side, without crossing the midline. Notably, a unilateral headache may just affect the frontal, temporal, or occipital regions of the head rather than the entire right or left side.

*Time to onset*: temporal relation between occurring new headaches/worsening of pre-existing headaches and exposure to the vaccine/infection. Headaches attributed to the occasional use of non-headache medication usually develop within minutes to hours of intake (8.1.9 Headache attributed to occasional use of non-headache medication – ICHD-3). The corresponding value for headaches attributed to substance exposures is within 12 h of exposure (8.1.11 Headache attributed to use of or exposure to other substance – ICHD-3).

*Attack duration*: time from onset until termination of a headache attack, meeting criteria for a particular headache type/subtype.

*Headache (end) days:* number of days affected by headache for any part or the whole of the day.

#### 2.4.2. General definitions

A definite positive history of COVID-19 was defined as having positive microbiologic testing (48). The intensity of COVID-19 was categorized as patients who needed merely outpatient care (home quarantine), those who needed ward admission, and those who received intensive medical care. Based on the most prominent symptoms, COVID-19 manifestations were described as systemic, respiratory, gastrointestinal, and neurological (vertigo, olfactory dysfunction, seizures, altered mental status, stroke, etc.). Vaccine platforms were categorized as inactivated (Sinopharm, Baharat, Barekat, Noora, and Fakhra), vector vaccines (AstraZeneca and Sputnik V), protein subunits (Spikogen, PastoCovac, and Razi-CovPars), and mRNA vaccines (Pfizer/BioNTech and Moderna) (49). Fever was defined as a morning oral temperature of >37.2°C or an afternoon temperature of >37.7°C (50).

### 2.5. Statistical analysis

Descriptive statistics were presented as mean ± standard deviation (SD) for numeric variables and as frequency (percentage) for categorical variables. The univariate logistic regression was used to evaluate the unadjusted impact of factors on the odds of outcomes. Moreover, age and sex were used to adjust the effect of factors on the odds of outcomes using multivariate logistic regression. All analyzes were conducted using SPSS (version 26) and R (version 4.2.1). A *p*-value less than 0.05 was considered statistically significant.

## 3. Results

### 3.1. Baseline, COVID-19-related, and vaccine-related characteristics of participants

**Figure 1:**
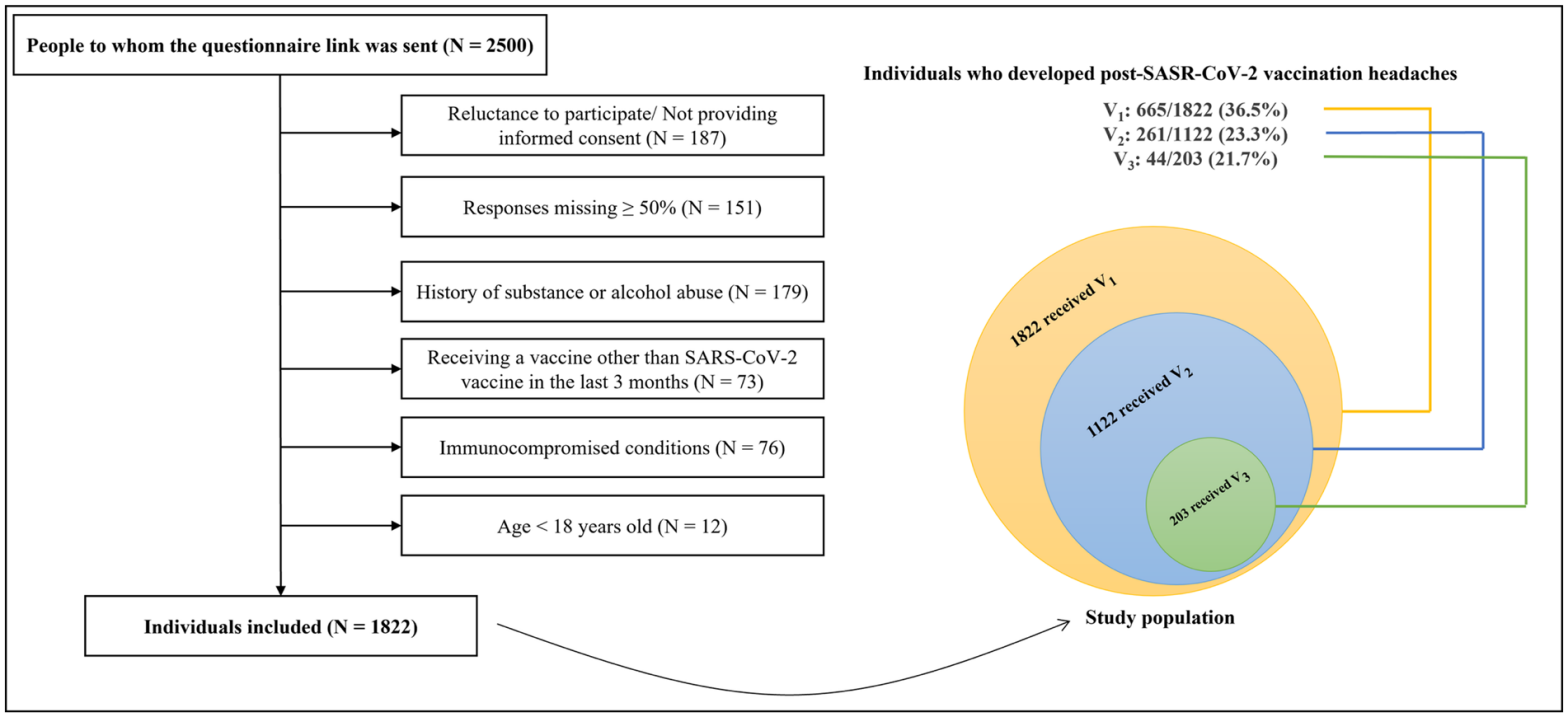
The flow diagram of participants. SARS-CoV-2, severe acute respiratory syndrome coronavirus 2; V1, 1st vaccine dose; V2, 2nd vaccine dose; V3, 3rd vaccine dose; N, number.

Figure 1 illustrates the participants’ flow diagram. The questionnaire link was randomly sent to 2,500 individuals, of whom 678 individuals did not meet the eligibility criteria for the following reasons: unwilling to participate or not providing informed consent (*N* = 187), a history of substance or alcohol abuse (*N* = 179), response missing ≥50% (*N* = 151), immunocompromised conditions (*N* = 76), receiving a vaccine other than SARS-CoV-2 during the last three months (*N* = 73), and age < 18 years old (*N* = 12). Eventually, 1822 individuals (mean age: 34.49 ± 11.09, female: 71.5%) were included. Among the 1822 responders, all of whom had received the 1st dose, of which 1,122 had received 2 doses, and 203 had received 3 doses, totaling 3,147 vaccination events (1st dose receivers: 1822, 2nd dose receivers: 1122, 3rd dose receivers: 203).

Table 1 indicates participants’ baseline characteristics. Most of the participants (68.3%) reported no remarkable past medical history. A history of controlled thyroid disorder was the most frequent comorbidity (4.4%). Previous history of headache disorders was reported by 52.0% of individuals, of whom 42.6 and 35.4% had migraine and TTH, respectively. A positive COVID-19 history was reported by 42.8%. Of 3,147 total administered vaccine doses, Sinopharm (38.5%), AstraZeneca (26.6%), and Sputnik-V (25.3%) were the most injected vaccines. Post-vaccination fever was reported by 29.2% of participants. Supplementary Tables S4, S5 provide information about other COVID-19-related and vaccine-related characteristics.

**Table 1:** Participants’ baseline and COVID-19 related characteristics (*N* = 1822).

Variable		
Age		34.49 ± 11.09
Sex	Female	1,303 (71.5)
	Male	510 (28.0)
Education (years)		16.0 [14.0, 18.0]
PMH^||^	Healthy	1,245 (68.3)
	Thyroid disorders	80 (4.4)
	Psychiatric^†^	52 (2.9)
	HTN	51 (2.8)
	Autoimmune	50 (2.7)
	Respiratory	44 (2.4)
	Cardiovascular^‡^	25 (1.4)
	DM	11 (0.6)
	Others	18 (1.0)
Primary headaches		947 (52.0)
Primary headaches type	Migraine	403 (42.6)
	Tension-type	335 (35.4)
	Cluster	29 (3.1)
	Other	180 (19.0)
History of COVID-19		780 (42.8)

Vaccine platforms	1st dose (1822)	2nd dose (1122)	3rd dose (203)	Total (3147)
Vector	1,033 (56.7)	498 (44.4)	100 (49.3)	1,631 (51.8)
Inactivated	714 (39.2)	579 (51.6)	60 (29.6)	1,353 (43.0)
mRNA	48 (2.6)	40 (3.6)	13 (6.4)	101 (3.2)
Protein subunits	2 (0.1)	2 (0.2)	26 (12.8)	30 (1.0)

Categorial data are presented as numbers (%). Symmetric numeric data are summarized by mean ± standard deviation (SD), and asymmetric numeric data are described using median [IQR]. IQR, interquartile range; PMH, past medical history; HTN, hypertension; DM, diabetes mellitus. ^†^Psychiatric disorders, including depressive disorders, anxiety disorders, trauma- and stress-related disorders. ^‡^Cardiovascular disorders except for HTN. ^||^Not including the previous history of headache disorders.

### 3.2. Headache characteristics attributed to COVID-19 infection

Table 2 shows headache characteristics following COVID-19 infection. Of 780 individuals with a history of COVID-19, 287 (36.8%) reported headaches attributed to COVID-19, of which 59.2 and 31.4% were migraine-like and TTH. The detailed characteristics of headaches attributed to COVID-19 infection are reported in Supplementary Result 1.

**Table 2:** Headaches characteristics attributed to COVID-19 infection and SARS-CoV-2 vaccination.

Variable		Post-COVID-19 headaches (*N* = 287)	Post-SARS-CoV-2 vaccination headaches (*N* = 970)^¶^
Primary headaches history		178 (62.0)	573 (59.1)
Primary headaches type	Migraine	61 (34.3)	244/573 (42.6)
	Tension-type	39 (21.9)	223/573 (38.9)
	Cluster	14 (7.9)	22/573 (3.8)
	Other	64 (36.0)	84/573 (14.7)
Similarity to primary headaches^§^		74/178 (41.6)	332/573 (57.9)
History of COVID-19 headaches		-	196 (20.2)
Similarity to COVID-19 headaches^§^		-	137/196 (69.9)
History of post-1st dose headaches		-	166/305^††^ (54.4)
Similarity to post-1st dose headaches^§^		-	107/166 (64.5)
History of post-2nd dose headaches		-	17/44^††^ (38.6)
Similarity to post-2nd dose headaches^§^		-	11/17 (64.7)
Headaches type	Migraine-like	170 (59.2)	350 (36.1)
	Tension-type	90 (31.4)	451 (46.5)
	Undifferentiated	27 (9.4)	169 (17.4)
Headaches intensity	Mild	35 (12.2)	187 (19.3)
	Moderate	143 (49.8)	495 (51.0)
	Severe	109 (38.0)	288 (29.7)
Headaches quality	Dull ache	84 (29.3)	129 (13.3)
	Sharp	62 (21.6)	153 (15.8)
	Pressing	97 (33.8)	527 (54.3)
	Pulsatile/throbbing	44 (15.3)	147 (15.2)
Headaches location	Frontal	48 (16.7)	144 (14.8)
	Temporal	34 (11.8)	121 (12.5)
	Occipital	13 (4.5)	40 (4.1)
	Top of the head	23 (8.0)	60 (6.2)
	Entire head	92 (32.1)	274 (28.2)
	Nuchal region^||^	0	3 (0.3)
	Entire head and nuchal region^||^	9 (3.1)	42 (4.3)
	Multiple^†^	68 (23.7)	270 (27.8)
Headaches lateralization	Unilateral/ more prominent on one side	85 (29.6)	294 (30.3)
	Bilateral	202 (70.4)	676 (69.7)
Time to onset (h)		24.0 [2.0, 24.0]	10.0 [4.0, 24.0]
Time to onset	<1 d	120 (41.8)	690 (71.1)
	1 ≤ onset<2 d	72 (25.1)	137 (14.1)
	2 ≤ onset<7 d	51 (17.8)	78 (8.0)
	≥7 d	2 (0.7)	35 (3.6)
Time to maximum intensity (d/h)		2.0 d [2.0, 4.0]	15.0 h [4.0, 24.0]
Attack duration (h)		3.0 [1.0, 5.0]	2.5 [2.0, 3.0]
Attack duration	<1 h	13 (4.5)	44 (4.5)
	1 ≤ duration<4 h	101 (35.2)	553 (57.0)
	4 ≤ duration<24 h	46 (16.0)	187 (19.3)
	24 ≤ duration<72 h	18 (6.3)	26 (2.7)
	≥72 h	34 (11.8)	41 (4.2)
Headaches end (d/h)		5 d [2.0, 10.0]	24.0 h [4.0, 48.0]
Headaches end	>48 h	27 (9.4)	555 (57.2)
	≥48 h	230 (80.1)	350 (36.1)
Analgesic use^‡^		246 (85.7)	824 (84.9)
Analgesic response	<50%	107 (43.5)	282/824 (34.2)
	≥50%	131 (53.3)	519/824 (63.0)

Categorial data are presented as numbers (%). Asymmetric numeric data are described using median [IQR]. IQR, interquartile range; COVID-19, coronavirus disease 2019; SARS-CoV-2, severe acute respiratory syndrome coronavirus 2; d, day(s); h, hour(s). ^†^At least two locations at the head or neck. ^‡^Using analgesics, such as acetaminophen, naproxen, ibuprofen, etc. for headaches. ^§^A general inquiry was used to subjectively assess the similarity between the patients’ post-vaccination headaches and their prior headaches, based on their own opinion, without specifically focusing on any particular characteristics. ^||^Nuchal region was defined as the posterior part of the upper neck, which includes the region where the neck muscles attach to the cranium. ^¶^Out of the 1822 respondents who had received the 1st dose, 1,122 had also received the 2nd dose, and 203 had received the 3rd dose, resulting in a total of 3,147 vaccination events. Among these individuals, 665, 261, and 44 recipients of the 1st, 2nd, and 3rd doses, respectively, experienced headaches, contributing to a total of 970 headache events within the 3,147 vaccination events. ^††^A history of headaches following the administration of the 1st dose was calculated among the recipients of the 2nd and 3rd doses (*N* = 305), and a history of headaches following the administration of the 2nd dose was calculated among the recipients of the 3rd doses (*N* = 44).

### 3.3. Headache characteristics attributed to SARS-CoV-2 vaccination

Table 2 shows the characteristics of headaches attributed to SARS-CoV-2 vaccination. Supplementary Table S6 and Supplementary Results 2 indicate the characteristics of headache attributed to SARS-CoV-2 vaccination for each different vaccine exposure (1st, 2nd, and 3rd SARS-CoV-2 vaccine dose).

The prevalence of headaches attributed to the 1st, 2nd, and 3rd dose of SARS-CoV-2 vaccination was 36.5% (665/1822 1st dose-receivers), 23.3% (261/1122 2nd dose-receivers), and 21.7% (44/203 3rd dose-receivers), respectively (*p* < 0.001). Considering the total vaccination events, we found a headache prevalence of 30.8% (970/3147 total vaccination events) following SARS-CoV-2 vaccination. A history of primary headaches and post-COVID-19 headaches were reported by 59.1 and 20.2% of patients who experienced post-vaccination headaches. More than half (54.4%) of the 2nd and 3rd dose-receivers who developed post-vaccination headaches had a history of headaches following the 1st dose, and 38.6% of the 3rd dose-receivers had a history of headaches following the 2nd dose. Objectively, 57.9, 69.9, and 64.5% of patients reported their post-vaccination headaches were similar to their primary headaches, post-COVID-19 headaches, and headaches following the previous vaccine doses, respectively.

Headaches attributed to SARS-CoV-2 vaccination were mostly TTH (46.5%), followed by migraine-like headaches (36.1%). They were mainly bilateral (69.7%) and moderate (51.0%), with a pressing quality (54.3%), affecting the entire head or multiple regions of the head and/or neck (56.0%). Headache onset was less than 2 days after vaccination in most of the participants (85.2%), with a median of 10 h after vaccination [4.0, 24.0] and the majority (71.1%) initiated in less than a day. Post-vaccination headaches reached their maximum intensity 15 h after vaccination [4.0, 24.0]. The median attack duration was 2.5 h [2.0, 3.0], with most of the patients (57.0%) reporting a duration between 1–4 h. Less than 7 % (6.9%) of the participants experienced attack durations of more than 24 h. The headaches ended 24 h after vaccination [4.0, 48.0], ranging from a half-hour to 21 days, with the majority ending in less than 48 h (57.2%). Of 84.9% of patients who used analgesics for their headaches, 63.0% responded more than 50%.

### 3.4. Factors associated with developing post-SARS-CoV-2 vaccination headaches

The unadjusted impact of variables on developing headaches following the 1st, 2nd, and 3rd doses are shown in Supplementary Table S7. The odds of headache slightly raised with each additional year of age (V_1_: OR = 1.02 [95% CI: 1.01, 1.02], *p* = 0.001; V_2_: OR = 1.03 [1.01, 1.05], *p* < 0.001). Furthermore, the female sex was associated with increased odds of post-SARS-CoV-2 vaccination headaches (V_1_: OR = 1.82 [1.45, 2.27], p < 0.001; V_2_: 1.67 [1.19, 2.33], *p* = 0.003). Table 3 indicates multivariate logistic regression results after adjusting the effect of each variable on the outcome for age and sex. Accordingly, the factors increasing the odds of post-SARS-CoV-2 vaccination headaches can be categorized as below:

(a) *Factors for which significant associations were observed for three doses*: headaches following the previous vaccine doses, vector vaccine platform, and post-vaccination fever.

**Table 3:** Adjusted impact of variables on the developing headaches following SARS-CoV-2 vaccination.

Variables	1st dose		2nd dose		3rd dose	
	OR (95% CI)	*P*	OR (95% CI)	*P*	OR (95% CI)	*P*
PMH						
Thyroid disorders	0.91 (0.57, 1.45)	0.701	1.00 (0.47, 1.95)	0.997	3.84 (0.66, 22.39)	0.119
Psychiatric disorders^†^	1.50 (0.85, 2.64)	0.159	0.76 (0.22, 2.00)	0.611	3.28 (0.78, 12.56)	0.085
HTN	1.43 (0.79, 2.62)	0.237	1.63 (0.58, 4.12)	0.322	1.86 (0.23, 11.22)	0.517
Autoimmune disorders	0.65 (0.34, 1.19)	0.174	0.79 (0.23, 2.13)	0.673	3.81 (0.70, 18.72)	0.097
Respiratory disorders	0.89 (0.46, 1.69)	0.738	1.86 (0.76, 4.18)	0.148	1.44 (0.20, 6.48)	0.665
Cardiovascular disorders^‡^	1.05 (0.45, 2.39)	0.906	1.28 (0.28, 4.39)	0.715	NA	NA
DM	1.40 (0.41, 4.96)	0.589	4.69 (0.75, 36.41)	0.096	3.31 (0.13, 86.94)	0.407
Primary headaches history	1.32 (1.08, 1.62)	0.007**	1.64 (1.15, 2.35)	0.007**	1.42 (0.71, 2.92)	0.328
Primary headaches type						
Migraine vs. Tension-type	0.80 (0.58, 1.10)	0.166	0.88 (0.53, 1.45)	0.604	1.76 (0.65, 4.78)	0.265
Cluster vs. Tension-type	0.61 (0.27, 1.33)	0.222	2.22 (0.91, 5.37)	0.078	1.77 (0.14, 22.60)	0.658
History of COVID-19 infection	1.08 (0.88, 1.33)	0.442	1.17 (0.84, 1.65)	0.358	1.56 (0.77, 3.26)	0.226
COVID-19 severity						
Ward admission vs. home quarantine	1.25 (0.63, 2.45)	0.517	3.26 (1.00, 10.27)	0.042*	1.69 (0.08, 18.99)	0.677
ICU admission vs. home quarantine	0.29 (0.01, 2.05)	0.276	1.49 (0.07, 16.70)	0.753	2.13 (0.09, 27.32)	0.567
COVID-19 manifestation						
Systemic vs. Respiratory	0.93 (0.65, 1.32)	0.674	1.16 (0.67, 1.97)	0.596	0.92 (0.31, 2.53)	0.873
Gastrointestinal vs. Respiratory	1.28 (0.84, 1.96)	0.245	1.02 (0.49, 2.00)	0.963	0.76 (0.19, 2.54)	0.670
Neurological vs. Respiratory	1.41 (0.80, 2.49)	0.232	1.20 (0.48, 2.73)	0.680	0.83 (0.12, 3.90)	0.829
History of COVID-19 headaches	1.06 (0.78, 1.46)	0.698	2.02 (1.26, 3.31)	0.004**	2.83 (1.17, 7.47)	0.026*
COVID-19 headaches type						
Migraine-like vs. Tension-type	1.40 (0.82, 2.42)	0.224	1.45 (0.74, 2.92)	0.287	4.15 (1.23, 16.99)	0.030*
Undifferentiated vs. Tension-type	1.33 (0.54, 3.24)	0.529	1.29 (0.41, 3.70)	0.649	0.94 (0.04, 9.20)	0.958
COVID-19 headaches intensity						
Moderate vs. Mild	1.56 (0.68, 3.85)	0.314	5.05 (1.37, 32.81)	0.036*	1.16 (0.21, 9.14)	0.872
Severe vs. Mild	1.95 (0.83, 4.90)	0.136	4.44 (1.17, 29.22)	0.056	2.37 (0.41, 19.43)	0.361
COVID-19 headaches time to onset						
<1 d vs. Manifesting	1.23 (0.58, 2.65)	0.595	1.01 (0.40, 2.62)	0.986	3.31 (0.61, 22.12)	0.183
1 ≤ onset<2 d vs. Manifesting	0.73 (0.34, 1.57)	0.420	0.50 (0.19, 1.36)	0.176	0.43 (0.08, 2.16)	0.303
2 ≤ onset<7 d vs. Manifesting	0.59 (0.25, 1.36)	0.218	1.17 (0.44, 3.14)	0.750	0.17 (0.01, 1.49)	0.152
≥7 d vs. Manifesting	1.12 (0.04, 29.99)	0.941	2.05 (0.07, 57.74)	0.633	NA	0.991
COVID-19 headaches attack duration	1.00 (0.97, 1.02)	0.899	1.03 (1.00, 1.06)	0.031*	1.01 (0.97, 1.05)	0.685
COVID-19 headaches attack duration, categorical						
1 ≤ duration<4 h vs. <1 h	1.06 (0.33, 3.75)	0.921	2.78 (0.48, 52.99)	0.348	0.90 (0.07, 21.88)	0.938
4 ≤ duration<24 h vs. <1 h	1.48 (0.42, 5.61)	0.547	4.34 (0.69, 85.08)	0.188	0.61 (0.04, 16.88)	0.729
24 ≤ duration<72 h vs. <1 h	0.97 (0.22, 4.41)	0.964	4.42 (0.56, 94.15)	0.213	1.03 (0.05, 34.30)	0.986
≥72 h vs. <1 h	1.30 (0.35, 5.22)	0.697	3.04 (0.43, 61.81)	0.333	1.00 (0.06, 28.82)	0.998
COVID-19 headaches end	1.04 (1.01, 1.07)	0.010*	1.01 (0.98, 1.03)	0.626	1.02 (0.93, 1.12)	0.689
COVID-19 headache end, categorical						
≥48 h vs. <48 h	3.20 (1.28, 9.29)	0.020*	4.32 (1.13, 29.16)	0.065	2.68 (0.38, 53.96)	0.389
History of post-1st dose headaches	-	-	30.52 (19.29,50.15)	<0.001***	3.78 (1.80, 7.96)	<0.001***
Post-1st dose headaches type						
Migraine-like vs. Tension-type	-	-	2.10 (1.20, 3.73)	0.010*	2.50 (0.67, 9.79)	0.177
Undifferentiated vs. Tension-type	-	-	0.39 (0.17, 0.88)	0.026*	0.60 (0.03, 5.95)	0.685
Post-1st dose headaches intensity						
Moderate vs. Mild	-	-	0.93 (0.43, 2.01)	0.849	0.77 (0.13, 4.82)	0.774
Severe vs. Mild	-	-	2.75 (1.16, 6.73)	0.023*	1.60 (0.23, 12.13)	0.632
Post-1st dose headaches intensity						
Severe vs. Non-severe	-	-	2.91 (1.50, 5.81)	0.002**	1.94 (0.47, 8.56)	0.362
Post-1st dose headaches time to onset						
1 ≤ onset<2 d vs. <1 d	-	-	0.69 (0.33, 1.44)	0.321	0.40 (0.04, 2.75)	0.372
2 ≤ onset<7 d vs. <1 d	-	-	1.72 (0.34, 12.56)	0.532	0.86 (0.03, 24.76)	0.919
≥7 d vs. <1 d	-	-	3.06 (0.48, 59.41)	0.313	NA	0.994
Post-1st dose headaches attack duration	-	-	1.01 (0.95, 1.07)	0.846	1.01 (0.93, 1.09)	0.840
Post-1st dose headaches attack duration, categorical						
1 ≤ duration<4 h vs. <1 h	-	-	3.65 (0.97, 17.62)	0.070	NA	NA
4 ≤ duration<24 h vs. <1 h	-	-	4.36 (1.07, 22.26)	0.050	NA	NA
24 ≤ duration<72 h vs. <1 h	-	-	1.98 (0.25, 16.24)	0.510	NA	NA
≥72 h vs. <1 h	-	-	2.13 (0.40, 13.43)	0.391	NA	NA
Post-1st dose headaches end	-	-	1.00 (0.99, 1.01)	0.589	1.11 (0.88, 1.40)	0.376
Post-1st dose headaches end, categorical						
≥48 h vs. >48 h	-	-	2.01 (0.85, 5.12)	0.123	NA	NA
History of post-2nd dose headaches	-	-	-	-	12.41 (4.73, 35.88)	<0.001***
Post-2nd dose headaches type						
Migraine-like vs. Tension-type	-	-	-	-	1.66 (0.26,12.41)	0.596
Undifferentiated vs. Tension-type	-	-	-	-	NA	0.995
Post-2nd dose headaches intensity						
Moderate vs. Mild	-	-	-	-	2.44 (0.24, 27.92)	0.449
Severe vs. Mild	-	-	-	-	2.72 (0.21, 45.38)	0.445
Post-2nd dose headaches intensity						
Severe vs. Non-severe	-	-	-	-	1.50 (0.20, 14.75)	0.699
Post-2nd dose headaches end	-	-	-	-	1.01 (0.99, 1.03)	0.538
Post-2nd dose headaches end, categorical						
≥48 h vs. >48 h	-	-	-	-	4.00 (0.42, 53.76)	0.245
Vaccine platform^§^						
Vector vs. Inactivated	3.88 (3.07, 4.92)	<0.001***	2.44 (1.70, 3.52)	<0.001***	4.34 (1.78, 12.29)	0.002**
Protein Subunit vs. Inactivated	3.70 (0.14, 96.83)	0.364	7.11 (0.28, 182.43)	0.169	1.79 (0.42, 7.00)	0.404
mRNA vs. Inactivated	0.94 (0.43, 1.89)	0.865	1.51 (0.58, 3.43)	0.360	0.00 (0.00, 1.00)	0.989
Analgesics use before vaccination^||^	1.31 (0.97, 1.75)	0.074	2.36 (1.42, 3.84)	<0.001***	1.19 (0.32, 3.66)	0.777
Post-vaccination fever^¶^	4.72 (3.79, 5.90)	<0.001***	6.85 (4.68, 10.10)	<0.001***	9.74 (4.56, 22.10)	<0.001***

Adjusted with age and sex. OR, odds ratio; CI, confidence interval; P, probability value; COVID-19, coronavirus disease 2019; SARS-CoV-2, severe acute respiratory syndrome coronavirus 2; M, male; F, female; PMH, past medical history; HTN, hypertension; DM, diabetes mellitus; ICU, intensive care unit; NA, not applicable. ^†^Psychiatric disorders, including depressive disorders, anxiety disorders, trauma- and stress-related disorders. ^‡^Cardiovascular disorders except for HTN. ^§^The effect of the vaccine platform on post-SARS-CoV-2 headaches is reported only for headaches following the same vaccine dose. ^||^The effect of using analgesics before vaccination on post-SARS-CoV-2 headaches is reported only for headaches following the same vaccine dose. ^¶^The effect of post-vaccination fever on post-SARS-CoV-2 headaches is reported only for headaches following the same vaccine dose. ^*^Significant at *p* < 0.05. ^**^Significant at *p* < 0.001. ^***^Significant at *p* < 0.001.

Having a headache after each dose of the vaccine was a strong predictor of headache occurrence following the next vaccine doses; post-1st dose headaches increased the odds of post-2nd dose and 3rd dose headaches by 30.52 ([95% CI: 19.29, 50.1], *p* < 0.001) and 3.78 times ([1.80, 7.96], *p* < 0.001). Post-2nd dose headaches increased the odds of post-3rd dose headaches by 12.41 ([4.73, 35.88], *p* < 0.001). Vector vaccines, compared to inactivated ones, significantly increased the odds of post-SARS-CoV-2 vaccination headaches (V_1_: aOR = 3.88 [3.07, 4.92], *p* < 0.001; V_2_: aOR = 2.44 [1.70, 3.52], *p* < 0.001; V_3_: aOR = 4.34 [1.78, 12.29], *p* = 0.002). Patients who developed a fever after vaccination had significantly increased odds of post-SARS-CoV-2 vaccination headaches (V_1_: aOR = 4.72 [3.79, 5.90], *p* < 0.001; V_2_: aOR = 6.85 [4.68, 10.10], *p* < 0.001; V_3_: aOR = 9.74 [4.56, 22.10], *p* < 0.001).

(b) *Factors for which significant associations were observed for two doses:* history of primary headaches and post-COVID-19 headaches.

Patients with a previous history of primary headaches had increased odds of post-1st dose and 2nd dose headaches (V_1_: aOR = 1.32 [1.08, 1.62], *p* = 0.007; V_2_: aOR = 1.64 [1.15, 2.35], *p* = 0.007). However, the type of primary headaches did not significantly affect post-vaccination headaches odds. A history of developing headaches following COVID-19, increased the odds of post-2nd dose and 3rd dose headaches (V_2_: aOR = 2.02 [1.26, 3.31], *p* = 0.004; V_3_: aOR = 2.83 [1.17, 7.47], *p* = 0.026).

(c) *Factors for which significant associations were observed for one dose:* COVID-19 severity, some characteristics of post-COVID-19 headaches (migraine-like, moderate intensity, longer attack durations, longer days of having headaches), and some characteristics of headaches after the previous vaccine dose (migraine-like, severe).

Although a history of COVID-19 did not significantly affect post-vaccination headaches odds, compared to patients who were quarantined at home, those who were hospitalized in the ward had higher odds of developing post-2nd dose headaches (aOR = 3.26 [1.00, 10.27], *p* = 0.042). Individuals whose COVID-19 headache was migraine-like, compared to TTH, had significantly higher odds of developing post-3rd dose headaches (aOR = 4.15 [1.23, 16.99], *p* = 0.030). Furthermore, compared to the mild intensity, post-COVID-19 headaches with moderate intensity increased the odds of post-2nd dose headache (aOR = 5.05 [1.37, 32.81], *p* = 0.036). Furthermore, each hour of increased duration of post-COVID-19 headache attacks slightly increased the odds of post-2nd dose headaches (aOR = 1.03 [1.00, 1.06], *p* = 0.031), and each day that the COVID-19 headache lasted longer slightly increased the likelihood of post-1st dose headaches (aOR = 1.04 [1.01, 1.07], *p* = 0.010). People whose headaches following COVID-19 lasted more than 48 h were 3.2 times ([1.28, 9.29], *p* = 0.020) more likely to develop post-1st dose headaches. Individuals with migraine-like post-1st dose headaches had increased odds of developing post-2nd dose headaches (aOR = 2.10 [1.20, 3.73], p = 0.010). Furthermore, patients with severe post-1st dose headaches, compared to non-severe headaches, had increased odds of post-2nd dose headaches (aOR: 2.91 [1.50, 5.81], *p* = 0.002).

**Figure 2:**
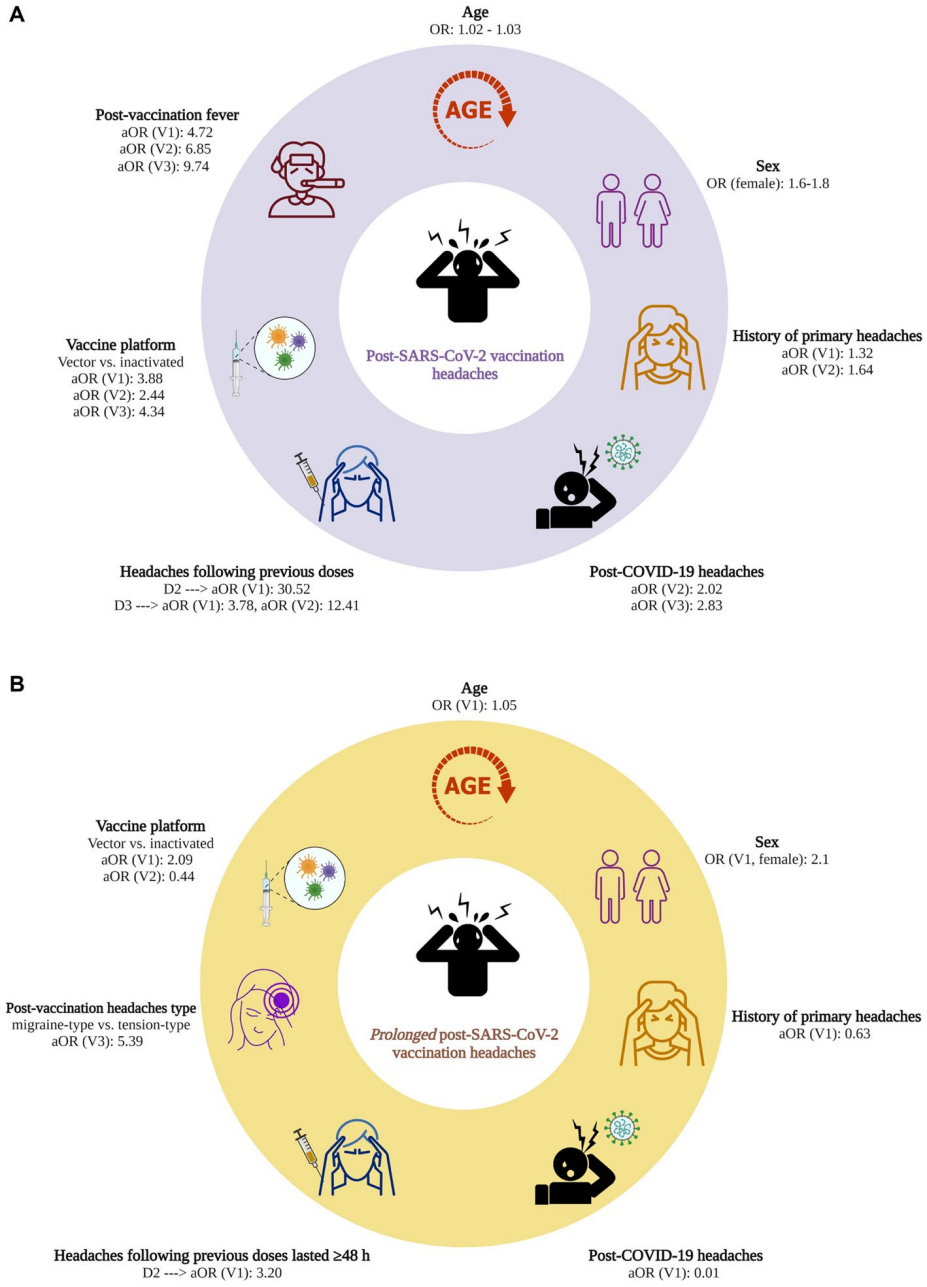
**(A)** Factors associated with post-SARS-CoV-2 vaccination headaches. **(B)** Factors associated with prolonged (≥ 48 h) post-SARS-CoV-2 vaccination headaches. OR, odds ratio; aOR, adjusted odds ratio; COVID-19, coronavirus disease 2019; SARS-CoV-2, severe acute respiratory syndrome coronavirus 2; V1, 1st vaccine dose; V2, 2nd vaccine dose; V3, 3rd vaccine dose; Created with BioRender.com.

Figure 2A illustrates the factors associated with developing post-SARS-CoV-2 vaccination headaches, for which significant associations were observed for at least two vaccine doses.

### 3.5. Factors associated with developing prolonged (≥ 48 h) post-SARS-CoV-2 vaccination headaches

Supplementary Table S8 shows the unadjusted impact of variables on developing prolonged post-SARS-CoV-2 vaccination headaches. Each year of increased age slightly increased the odds of prolonged post-1st dose headaches (OR = 1.05 [1.03, 1.07], *p* < 0.001). Being female was also associated with increased odds of prolonged post-1st dose headache (OR = 2.13 [1.33, 3.45], *p* = 0.002). Table 4 displays multivariate logistic regression results after adjusting the effect of each variable on the outcome for age and sex. Accordingly, the factors associated with developing prolonged post-SARS-CoV-2 vaccination headaches can be categorized as below:

(a) *Factors increasing the odds of prolonged post-vaccination headaches* (associations were observed only for one dose): psychiatric disorders, prolonged headaches after the previous dose, and migraine-like headaches at the same dose.

**Table 4:** Adjusted impact of variables on developing prolonged headache following SARS-CoV-2 vaccination.

Variables	1st dose		2nd dose		3rd dose	
	OR (95% CI)	*P*	OR (95% CI)	*P*	OR (95% CI)	*P*
PMH						
Thyroid disorders	0.51 (0.20, 1.18)	0.137	0.99 (0.28, 3.67)	0.987	NA	NA
Psychiatric disorders ^†^	2.58 (1.05, 6.45)	0.039*	0.83 (0.10, 7.15)	0.856	1.02 (0.09, 11.14)	0.989
HTN	0.77 (0.30, 1.92)	0.583	0.98 (0.19, 5.43)	0.976	NA	NA
Autoimmune disorders	1.14 (0.37, 3.22)	0.809	2.71 (0.33, 56.24)	0.398	1.55 (0.12, 38.00)	0.741
Respiratory disorders	1.05 (0.28, 3.29)	0.937	0.54 (0.11, 2.33)	0.418	0.78 (0.03, 22.33)	0.869
Cardiovascular disorders^‡^	2.64 (0.56, 13.77)	0.214	NA	NA	NA	NA
DM	0.53 (0.07, 2.94)	0.487	0.36 (0.02, 4.00)	0.416	NA	NA
Other	0.39 (0.06, 1.72)	0.258	NA	NA	NA	NA
Primary headaches history	0.63 (0.44, 0.90)	0.010*	0.99 (0.49, 1.98)	0.984	1.50 (0.40, 5.73)	0.548
Primary headaches type						
Migraine vs. Tension-type	0.80 (0.47, 1.34)	0.392	1.03 (0.41, 2.56)	0.954	0.43 (0.06, 2.53)	0.363
Cluster vs. Tension-type	NA	NA	0.77 (0.17, 3.43)	0.729	NA	NA
History of COVID-19 infection	0.97 (0.68, 1.38)	0.847	0.93 (0.48, 1.79)	0.817	0.89 (0.22, 3.49)	0.872
COVID-19 severity						
Ward admission vs. home quarantine	0.92 (0.29, 2.64)	0.876	1.68 (0.18, 36.62)	0.672	NA	NA
ICU admission vs. home quarantine	NA	NA	NA	NA	NA	NA
COVID-19 manifestation						
Systemic vs. Respiratory	0.96 (0.49, 1.83)	0.893	1.89 (0.66, 5.58)	0.239	2.82 (0.20, 91.88)	0.475
Gastrointestinal vs. Respiratory	1.56 (0.75, 3.18)	0.226	0.87 (0.21, 3.57)	0.849	0.23 (0.01, 2.40)	0.255
Neurological vs. Respiratory	1.00 (0.37, 2.54)	0.995	0.31 (0.04, 1.69)	0.200	NA	0.995
History of COVID-19 headaches	0.01 (0.00, 0.05)	<0.001***	0.95 (0.38, 2.36)	0.912	1.82 (0.29, 12.65)	0.525
COVID-19 headaches type						
Migraine-like vs. Tension-type	NA	NA	1.49 (0.40, 5.49)	0.545	1.23 (0.12, 12.86)	0.855
Undifferentiated vs. Tension-type	NA	NA	0.24 (0.01, 2.07)	0.242	NA	NA
COVID-19 headaches intensity						
Moderate vs. Mild	NA	NA	NA	NA	0.93 (0.03, 30.48)	0.962
Severe vs. Mild	NA	NA	NA	NA	2.93 (0.07, 131.56)	0.543
COVID-19 headaches time to onset						
<1 d vs. Manifesting	NA	NA	0.95 (0.16, 5.53)	0.958	1.15 (0.09, 15.64)	0.912
1 ≤ onset<2 d vs. Manifesting	NA	NA	0.52 (0.07, 3.43)	0.499	4.52 (0.25, 153.70)	0.329
2 ≤ onset<7 d vs. Manifesting	NA	NA	0.81 (0.12, 5.39)	0.824	NA	NA
≥7 d vs. Manifesting	NA	NA	NA	NA	NA	NA
COVID-19 headaches attack duration	0.33 (0.00, 0.97)	0.497	0.99 (0.95, 1.03)	0.490	1.03 (0.97, 1.13)	0.392
COVID-19 headaches end	NA	NA	1.02 (0.97, 1.09)	0.450	NA	NA
History of post-1st dose headaches	-	-	0.68 (0.25, 1.70)	0.413	0.75 (0.20, 2.74)	0.666
Post-1st dose headaches type						
Migraine-like vs. Tension-type	0.81 (0.54, 1.21)	0.303	1.91 (0.90, 4.13)	0.096	0.28 (0.01, 2.99)	0.329
Undifferentiated vs. Tension-type	0.68 (0.41, 1.11)	0.130	1.53 (0.43, 5.87)	0.514	NA	0.996
Post-1st dose headaches intensity						
Moderate vs. Mild	-	-	1.09 (0.30, 3.89)	0.899	2.78 (0.17, 83.61)	0.487
Severe vs. Mild	-	-	1.34 (0.37, 4.94)	0.653	1.79 (0.08, 68.27)	0.721
Post-1st dose headaches intensity						
Severe vs. Non-severe	-	-	1.26 (0.52, 3.13)	0.606	0.82 (0.08, 8.63)	0.864
Post-1st dose headaches time to onset						
1 ≤ onset<2 d vs. <1 d	-	-	1.66 (0.61, 4.85)	0.331	NA	NA
2 ≤ onset<7 d vs. <1 d	-	-	2.72 (0.32, 56.76)	0.399	NA	NA
≥7 d vs. <1 d	-	-	3.69 (0.51, 74.42)	0.254	NA	NA
Post-1st dose headaches attack duration	-	-	0.99 (0.91, 1.05)	0.680	1.07 (0.88, 1.39)	0.521
Post-1st dose headaches attack duration, categorical						
1 ≤ duration<4 h vs. <1 h	-	-	1.69 (0.15, 37.95)	0.678	NA	NA
4 ≤ duration<24 h vs. <1 h	-	-	2.64 (0.22, 60.98)	0.451	0.42 (0.00, 25.53)	0.673
24 ≤ duration<72 h vs. <1 h	-	-	0.96 (0.02, 39.50)	0.979	5.19 (0.03, 4982.95)	0.521
≥72 h vs. <1 h	-	-	4.03 (0.24, 127.13)	0.353	NA	NA
Post-1st dose headaches end	-	-	1.01 (1.00, 1.02)	0.203	2.79 (1.10, 25.39)	0.153
Post-1st dose headaches end, categorical						
≥48 h vs. >48 h	-	-	3.10 (1.08, 10.31)	0.045*	NA	NA
History of post-2nd dose headaches	-	-	-	-	1.36 (0.36, 5.53)	0.652
Post-2nd dose headaches type						
Migraine-like vs. Tension-type	-	-	1.67 (0.85, 3.33)	0.141	1.02 (0.12, 8.51)	0.985
Undifferentiated vs. Tension-type	-	-	0.70 (0.23, 2.07)	0.524	NA	NA
Post-2nd dose headaches intensity						
Moderate vs. Mild	-	-	-	-	0.37 (0.01, 6.88)	0.512
Severe vs. Mild	-	-	-	-	0.99 (0.03, 26.41)	0.994
Post-2nd dose headaches intensity						
Severe vs. Non-severe	-	-	-	-	2.03 (0.20, 25.41)	0.554
Post-2nd dose headaches attack duration						
1 ≤ duration<4 h vs. <1 h	-	-	-	-	NA	NA
4 ≤ duration<24 h vs. <1 h	-	-	-	-	0.15 (0.00, 3.83)	0.290
24 ≤ duration<72 h vs. <1 h	-	-	-	-	NA	0.998
≥72 h vs. <1 h	-	-	-	-	NA	0.997
Post-2nd dose headaches end	-	-	-	-	1.10 (1.01, 1.34)	0.161
Post-2nd dose headaches end, categorical						
≥48 h vs. >48 h	-	-	-	-	8.74 (0.52, 355.26)	0.171
Post-3rd dose headaches type						
Migraine-like vs. Tension-type	-	-	-	-	5.39 (1.15, 32.47)	0.043*
Undifferentiated vs. Tension-type	-	-	-	-	2.60 (0.08, 82.92)	0.544
Vaccine platform^§^						
Vector vs. Inactivated	2.09 (1.33, 3.37)	0.002**	0.44 (0.21, 0.89)	0.024*	0.49 (0.06, 3.24)	0.474
Protein Subunit vs. Inactivated	NA	0.982	NA	0.986	0.46 (0.02, 7.44)	0.584
mRNA vs. Inactivated	0.40 (0.02, 2.27)	0.391	0.35 (0.06, 1.76)	0.202	NA	NA
Analgesics use before vaccination^||^	2.12 (1.33, 3.38)	0.002**	2.08 (0.87, 5.39)	0.113	0.24 (0.01, 2.14)	0.243
Post-vaccination fever^¶^	1.18 (0.82, 1.70)	0.374	0.89 (0.47, 1.69)	0.731	0.58 (0.12, 2.46)	0.470

Adjusted with age and sex. OR, odds ratio; CI, confidence interval; P, probability value; COVID-19, coronavirus disease 2019; SARS-CoV-2, severe acute respiratory syndrome coronavirus 2; M, male; F, female; PMH, past medical history; HTN, hypertension; DM, diabetes mellitus; ICU, intensive care unit; NA, not applicable. ^†^Psychiatric disorders, including depressive disorders, anxiety disorders, trauma- and stress-related disorders. ^‡^Cardiovascular disorders except for HTN. ^§^The effect of the vaccine platform on post-SARS-CoV-2 headaches is reported only for headaches following the same vaccine dose. ^||^The effect of using analgesics before vaccination on post-SARS-CoV-2 headaches is reported only for headaches following the same vaccine dose. ^¶^The effect of post-vaccination fever on post-SARS-CoV-2 headaches is reported only for headaches following the same vaccine dose. ^*^Significant at *p* < 0.05. ^**^Significant at *p* < 0.001. ^***^Significant at *p* < 0.001.

Patients with a history of psychiatric disorders [defined as depressive disorders, anxiety disorders, trauma- and stress-related disorders (51)] had increased odds of prolonged post-1st dose headaches (aOR: 2.58 [1.05, 6.45], *p* = 0.039). Prolonged post-1st dose headaches significantly increased the odds of prolonged post-2nd dose headaches (aOR = 3.10 [1.08, 10.31], *p* = 0.045). The odds of prolonged post-3rd dose headaches was significantly increased in patients who experienced migraine-like, compared to the TTH, after receiving the 3rd dose (aOR = 5.39 [1.15, 32.47], *p* = 0.043).

(b) *Factors reducing the odds of prolonged post-vaccination headaches* (associations were observed only for one dose): history of primary headaches and post-COVID-19 headaches.

Having a history of primary headaches reduced the odds of prolonged post-1st dose headaches (aOR = 0.63 [0.44, 0.90], *p* = 0.010). Similarly, a history of developing headaches following COVID-19 significantly reduced the odds of prolonged post-1st dose headaches (aOR = 0.01 [0.00, 0.05], *p* < 0.001).

(c) *Factors with inconsistent effects on the odds of prolonged post-vaccination headaches:* vaccine platform

Compared to the inactivated vaccine platforms, vector platforms significantly increased the odds of prolonged post-1st dose headaches (aOR = 2.09 [1.33, 3.37], *p* = 0.002). Nevertheless, they reduced the odds of post-2nd dose headaches (aOR = 0.44 [0.21, 0.89], *p* = 0.024).

Figure 2B displays the factors significantly associated with prolonged post-vaccination headaches.

## 4. Discussion

**Figure 3:**
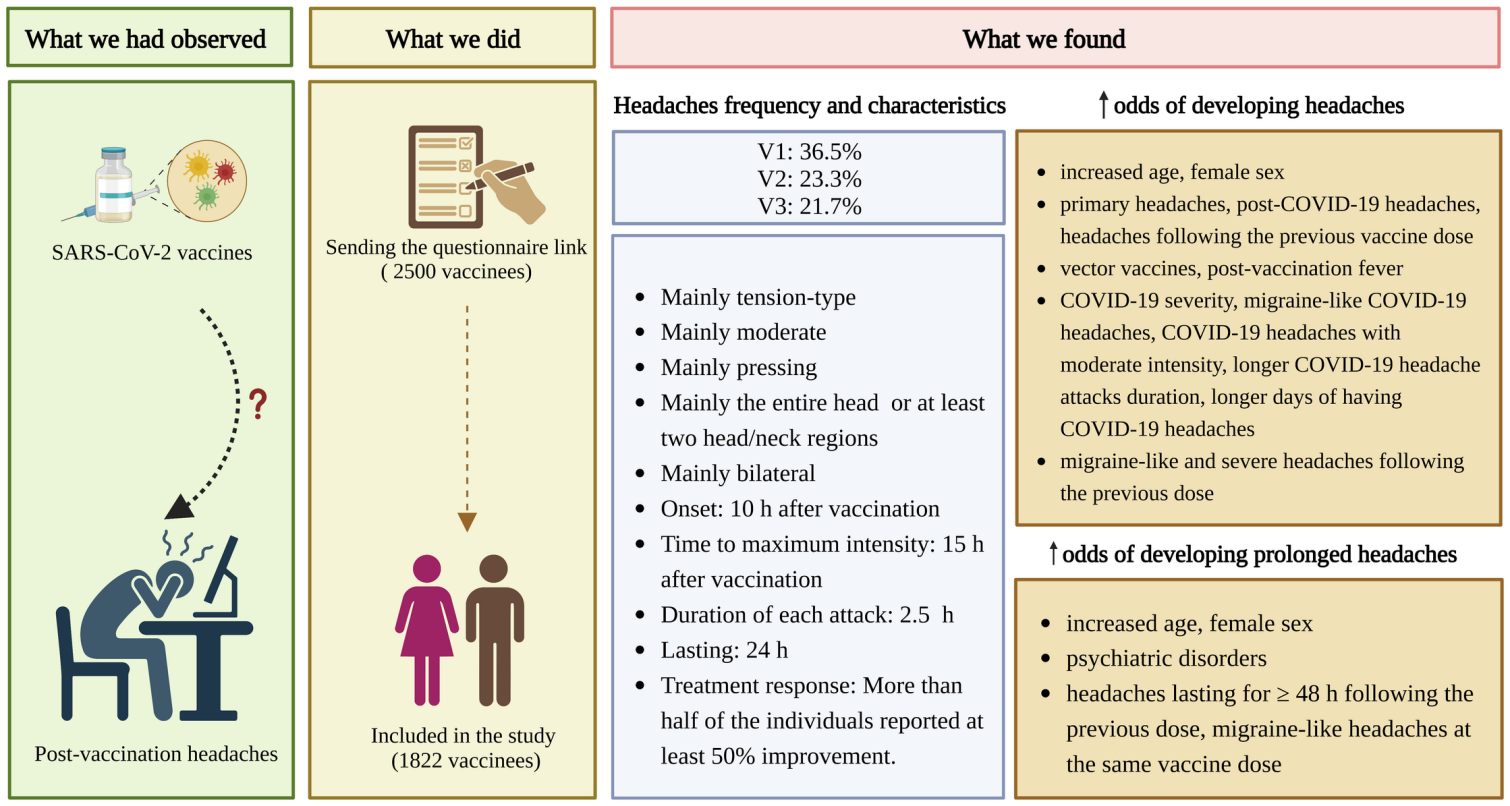
Research summary. COVID-19, coronavirus disease 2019; SARS-CoV-2, severe acute respiratory syndrome coronavirus 2; V1, 1st vaccine dose; V2, 2nd vaccine dose; V3, 3rd vaccine dose; h, hour; Created with BioRender.com.

Figure 3 provides an overview of the study with the key findings. We found a total post-SARS-CoV-2 vaccination headache prevalence of 30.8%. The occurrence of post-vaccination headaches decreased with increased exposure (36.5, 23.3, and 21.7% following the 1st, 2nd, and 3rd dose, respectively). More than half (59.1%) of the individuals who developed post-SASR-CoV-2 vaccination headaches reported having primary headaches. Similarity between post-vaccination headaches and primary headaches, post-COVID-19 headaches, and headaches following the previous doses was reported by 57.9, 69.9, and 64.5% of individuals, respectively. Headaches were mostly TTH (46.5%). Headaches were usually moderate (51.0%), bilateral (69.7%), pressing (54.3%), and responsive to analgesics (63.0%), affecting the entire head or multiple regions of the head/neck (56.0%). They usually started 10 h after vaccination, reached their maximum intensity 15 h after vaccination, and ended 24 h later. Each attack duration was nearly 2.5 h. Initiating headaches≥ two days after vaccination and attack durations of≥24 h were not common.

Increased age, being female, primary headaches, post-COVID-19 headaches, headaches following previous doses, vector vaccines, and post-vaccination fever increased post-SARS-CoV-2 vaccination headache odds. Other possible associated factors were COVID-19 severity, COVID-19 headaches characteristics (migraine-like, moderate intensity, longer attack duration, and longer days of COVID-19 headaches), and some headaches characteristics after the previous dose (migraine-like and severe headaches). Primary headaches and post-COVID-19 headaches reduced the odds of prolonged post-vaccination headaches. Increased age, being female, psychiatric disorders, prolonged headaches following the previous dose, and migraine-like headaches at the same dose may increase prolonged post-vaccination headaches odds. The effect of vector platforms on prolonged post-vaccination headaches requires further investigation. Notably, the observation that significant associations between specific variables and outcome measures did not apply to all three doses might reflect that vaccine dose has shown to be one of the strongest factors associated with vaccination AEs (52).

### 4.1. Prevalence and characteristics of headaches attributed to SARS-CoV-2 vaccination

Table 5 provides an overview of the literature related to post-SARS-CoV-2 vaccination headaches. A meta-analysis by Castaldo et al. on nearly 1.57 million vaccine receivers suggested that SARS-CoV-2 vaccines were associated with a two-fold increased risk of developing headaches within 7 days from the injection (19). The authors found a post-1st dose and 2nd dose headaches prevalence of 22 and 29%, respectively (19), which accords with our findings. Although we found that the occurrence of post-vaccination headaches decreased with increased exposure, to our knowledge, there is no clear evidence as to how the occurrence of post-vaccination headaches alters with further doses. According to Sekiguchi et al. cross-sectional study, the incidence of headaches was significantly higher after the 2nd dose compared to the 1st (21). However, this study was conducted on patients with a history of headaches, and it is not clear if the same applies to the general population (21). Further research is necessary to provide a comprehensive response to this inquiry. Consistent with our findings, Ekizoglu et al. study found a post-vaccination frequency of 30.6% (29). Another cross-sectional study among hospital health workers reported the headache prevalence of 48.8 and 33.5% after the 1st and 2nd dose (53), higher than what we observed. This difference can be justified by different study populations (healthcare workers vs. the general population). Silvestro et al. study on 841 migraine participants found higher post-vaccination headaches prevalence of 66.5 and 60.2% after the 1st and 2nd dose (23), supporting the idea that primary headaches are linked to an increased risk of developing headaches following SARS-CoV-2 vaccination.

**Table 5:** Overview of literature related to the post-SARS-CoV-2 vaccination headaches.

Author, year	Country	Study population	Vaccine type	Post-vaccination headache frequency	Characteristics and clinicodemographic associations of post-vaccination headaches
Jameie et al., (2023) (15) (This study)	Iran	The general population who voluntarily participated in the online survey	Mainly (90.4%) Sinopharm AstraZeneca Sputnik-v	After V_1_: 36.5% After V_2_: 23.3% After V_3_: 21.7%	More than half (59.1%) of the individuals with post-vaccination headaches had a history of primary headaches. Similarity to the primary headaches was reported by 57.9% of participants. Similarity to post-COVID-19 headaches was reported by 69.9% of participants. Similarity to headaches following the previous vaccine dose was reported by nearly 64.5% of participants. *Type*: mainly tension-type after V_1_ and V_2_ (47.7 and 46.7%, respectively) and migraine-like after V_3_ (65.9%) *Intensity*: mainly moderate (51.0%) *Quality*: mainly pressing (54.3%) *Location*: mainly the entire head (28.2%) or at least two regions of the head and neck (27.8%) *Lateralization*: mainly bilateral (69.7%) *Initiation*: 10 h [4.0, 24.0] after vaccination. In more than 70% of patients, the headaches initiate less than 24 h after vaccination. *Time to maximum intensity*: 15 h [4.0, 24.0] *Duration of each headache attack*: nearly 2.5 h [2.0, 3.0], between 1–4 h in more than half of patients (57.0%). *Lasting*: 24 h [4.0, 48.0]. However, 36.1% of individuals experienced headaches lasting ≥48 h. *Treatment response*: More than half of the individuals (63.5%) reported at least 50% improvement after using analgesics. *Factors increasing the odds of developing post-vaccination headaches*: Associations observed for at least two vaccine doses: increased age, female sex, history of primary headaches, history of post-COVID-19 headaches, history of headaches following the previous vaccine dose, vector vaccines, and post-vaccination fever. Associations observed for only one vaccine dose: COVID-19 severity, characteristics of COVID-19 headaches (migraine-like COVID-19 headaches, COVID-19 headaches with moderate intensity, longer headache attacks duration, and longer days of having COVID-19 headaches), and some characteristics of headaches after the previous vaccine dose (migraine-like and severe headaches following the previous dose). *Factors affecting the odds of developing prolonged (≥48 h) post-vaccination headaches (associations were observed for only one vaccine dose):* *Increasing the odds*: increased age, female sex, history of psychiatric disorders, history of headaches lasting for ≥48 h following the previous vaccine dose, migraine-like headaches at the same vaccine dose *Decreasing the odds*: history of primary headaches, history of post-COVID-19 headaches *Conflicting findings*: vector vaccines
Ceccardi et al. (2022) (13)	Italy	Individuals who visited the ED and were hospitalized due to a new or worsening headache in the 16 days following the COVID-19 vaccination	Pfizer-Comirnaty (predominant) Spikevax-Moderna Vaxzevria	ED headache admissions time-correlated to the COVID-19 vaccination: 10.8% of all ED headache admissions	*Type*: The headache most frequently reported by patients had migraine-like characteristics. *Intensity*: severe *Quality*: throbbing *Localization*: predominantly frontal or temporal *Accompanying symptoms*: nausea/vomiting and photo-phonophobia Over half -regardless of the final diagnosis- of hospitalized patients had a history of primary headaches.
Castaldo et al. (2022) (19)	NA	A systematic review of 84 papers (1.57 million participants)	BNT162b2 or ChAdOx1 (94%)	After V_1_: 22% After V_2_: 29% Placebo receivers: 10–12%	*Type, quality, accompanying symptoms*: In around one-third of the cases, headache has migraine-like features with pulsating quality, phono- and photophobia. In 40–60% of the cases, aggravation with activity was observed. *Initiation*: within the first 24 h No differences were detected across different vaccines or by mRNA-based vs. “traditional” ones. Most patients used some medication to treat headaches, the one perceived as the most effective being acetylsalicylic acid. The prevalence of headaches after the first injection of BNT162b2 was lower in older participants. Meta-regression analysis results did not find significantly different prevalence of post-vaccination headaches between men and women.
Sekiguchi et al. (2022) (21)	Japan	Nursing staff with a history of headache	COVID-19 mRNA vaccination	Migraine group: 69.2% Non-migraine group: 71.4% Healthy control: 37.9%	*Lateralization*: mostly bilateral (62.5–78.8%) *Initiation*: V_1_: 10 h, V_2_: 12 h *Lasting*: V_1_: 4.5 h, V_2_: 8 h The incidence of headaches was significantly higher after the second dose compared to the first (45.6% vs. 20.5%).
Ekizoglu et al. (2021) (29)	Turkey	Healthcare personnel	Mainly inactivated virus (CoronaVac)	30.6%	*Lateralization*: mostly bilateral *Accompanying symptoms*: without accompanying phenomena *Initiation*: 1 day [IQR: 0–2] *Lasting* ≥ 3 days: 25.9% Female dominance Less severe, and shorter than COVID-19-related headache The presence of primary headaches and migraine were significantly associated with COVID-19 vaccine-related headaches. Headaches during COVID-19 showed a significant association with headaches following the COVID-19 vaccine. Only thyroid diseases showed a significant association with vaccine-related headaches among the common comorbidities (HTN, DM, HLP, cardiac diseases, asthma).
Göbel et al. (2021) (30)	Germany UAE	Vaccinees at residential care homes	BNT162b2 mRNA	NA *Only patients with headaches were included in the study.	*Intensity*: moderate (46.2%), severe (32.1%), very severe (8.2%) *Quality*: pressing (49.2%), dull (40.7%) *Lateralization*: bilateral in 73.1% of the participants. *Location*: forehead (38.0%), temples (32.1%) *Initiation*: 18 h *Lasting*: 14 h Only 9.7% of those affected also report headaches resulting from previous vaccinations. In 66.6% of the participants, headache occurs as a single episode.
Göbel et al. (2021) (45)	Germany UAE	Vaccinees at residential care homes	ChAdOx1 nCoV-19 (AZD1222)	NA * Only patients with headaches were included in the study.	*Intensity*: severe (38.7%), moderate (35.2%), very severe (15.5%) *Quality*: pressing (50.4%), dull (37.7%) *Lateralization*: bilateral (75.8%) *Location*: forehead (40.0%) and temples (31.4%) *Initiation*: 14.5 h *Lasting*: 16.3 h
Mattiuzzi et al. (2021) (35)	Italy	Public Italian Medicines Agency (AIFA) database of Adverse Drug Reactions (RAM)	Pfizer Moderna AstraZeneca	The rate of headache/migraine episodes voluntarily reported by recipients of COVID-19 vaccines up to May 9, 2021: AstraZeneca:129/100,000 Pfizer: 103/100,000 Moderna: 21/100,000	The risk of developing headache/migraine episodes was the highest for recipients of the AstraZeneca vaccine, followed by those receiving the Pfizer vaccine. The number of voluntary reports for the Moderna COVID-19 vaccine was even lower than the daily frequency of headache disorders in Italy.
Silvestro et al. (2021) (23)	Italy	Migraine patients	Comirnaty Vaxzervria mRNA-1,273 Janssen	After V_1_: 66.47% After V_2_: 60.15%	Attacks following vaccination were referred to as more severe (50.62% of patients), long-lasting (52.80% of patients), and hardwearing (49.69% of patients) compared to the usually experienced migraine attacks. Over half of the patients perceived headache attacks as different from those usually experienced. Age, frequency of headache attacks, and previous COVID-19 infection were associated with an increased likelihood of experiencing headache attacks after the first COVID-19 vaccine administration. Correlation analysis showed a statistically significant correlation between the occurrence of headache attacks in the days following the first vaccine administration and the other systemic adverse reactions. The presence of a headache attack after the first vaccine administration was significantly associated with the occurrence of an attack after the second vaccine administration.

COVID-19, coronavirus disease 2019; SARS-CoV-2, severe acute respiratory syndrome coronavirus 2; ED, emergency department; HTN, hypertension; DM, diabetes mellitus; HLP, hyperlipidemia; V_1_, 1st vaccine dose; V_2_, 2nd vaccine dose; V_3_, 3rd vaccine dose; NA, not applicable.

In line with our findings, Ekizoglu et al. revealed that post-vaccination headaches are usually bilateral (29). Göbel et al. reported that post-ChAdOx1 nCoV-19 vaccination headaches are usually bilateral, with a pressing character (30). On the other hand, Ceccardi et al. study suggested different headaches characteristics following SARS-CoV-2 vaccination among patients who were admitted to the emergency department (ED) or hospitalized due to post-vaccination headaches; headaches were usually severe with a throbbing quality in this subgroup (13). Additionally, while we found TTH was the predominant headache type following the 1st and 2nd doses, their study indicated that migraine-like headache characteristics were reported by most patients (13), which could be a reflection of the different populations of their study (ED admission) compared to ours (general population). Our study revealed a frequency of 33.8 and 36.8% of migraine-like headaches after the 1st and 2nd doses. These results reflect those of Castaldo et al. meta-analysis, who also found migraine-like characteristics in about one-third of vaccinees who developed headaches (19).

Our finding broadly supports the work of other studies regarding the other characteristics of post-vaccination headaches; post-vaccination headaches usually initiate less than a day after vaccination, and delayed headache onset should be considered a red flag for serious conditions, such as vaccine-induced CVT (26). According to Ekizoglu et al. study, post-vaccination headaches initiated nearly 1 day [0–2] after vaccination (29). Consistently, Castaldo et al. meta-analysis showed that post-vaccination headaches are usually reversible, with onset within a few hours after the vaccination (19). Furthermore, we found that post-vaccination headaches occurred with a median of 10 h following vaccination, emphasizing that very early headache onset should also be investigated. Accordingly, previous studies have also highlighted that in patients with headaches beginning immediately after vaccination, physicians should be aware of other underlying causes, such as CVT (26). Notably, our study indicated that post-vaccination headaches responded well to analgesics in 63.0% of individuals, contrary to the existing knowledge about vaccine-induced CVT, which is usually treatment resistant (19).

### 4.2. Post-vaccination headaches resemblance to primary headaches and post-COVID-19 headaches

According to Göbel et al., post-vaccination headaches had a distinct phenotypic profile from primary headaches (30). Consistently, more than half of the migraineurs in the Silvestro et al. study reported their post-vaccination headaches were “different” from their primary headaches; they were more severe, long-lasting, and hardwearing (23). Notably, 57.9% of our study participants reported their post-vaccination headaches were “similar” to their primary headaches. This difference in findings may be due to the differences in the definition of our study (without focusing on any particular characteristics) with Silvestro et al. study (asking specifically about differences in intensity, duration, and response to painkillers), necessitating further investigation.

In our study, compared to the COVID-19 headaches, post-vaccination headaches initiated earlier (10 h after vaccination vs. 24 h after the infection) and lasted shorter (24 h after vaccination vs. 5 days after the infection). In alignment with our findings, Ekizoglu et al. study demonstrated that post-vaccination headaches are less severe and shorter than post-COVID-19 headaches (29).

### 4.3. Factors associated with developing headaches following SARS-CoV-2 vaccination

#### 4.3.1. Age and sex

Although in our study, the odds of headache following vaccination increased slightly with age, the odds of headache in the Silvestro et al. study slightly reduced with increased age, hence the necessity of further investigations in this regard. Similar to our findings, Ekizoglu et al. study suggested female dominance for post-vaccination headaches (29). Consistently, according to Al-Qazaz et al., women experienced significantly greater rates of severe and moderate systemic AEs, including headaches, following SARS-CoV-2 vaccination (54). Nevertheless, the results of a meta-regression analysis to address the effect of sex on post-vaccination headaches did not find a significantly different prevalence of post-vaccination headaches between men and women (19).

#### 4.3.2. Primary headaches

Consistent with the literature (13, 21, 29), we found a significant association between primary headaches and headaches following SARS-CoV-2 vaccination. A study by Sekiguchi et al. demonstrated post-vaccination headache frequency of 37.9% in individuals without a headache history, while 69.2 and 71.4% in those with a history of migraine and non-migraine headaches, respectively (21). Consistently, Ekizoglu et al. study identified that post-vaccination headaches occurred in 21.1% of those without a history of headaches, while 38.8% of those with a history of headaches (29). The authors indicated that the presence of primary headaches increased the odds of developing post-vaccination headaches (29).

#### 4.3.3. Post-COVID-19 headaches and headaches following the previous vaccine dose

Our results also reflect those of Silvestro et al., who indicated that developing headaches following COVID-19 increased the odds of developing post-vaccination headaches (23). Ekizoglu et al. also found a significant association between post-COVID-19 headaches and developing headaches following vaccination (29). Although the exact pathophysiology behind this correlation is not well understood, some evidence has suggested similar cytokine-mediated pathomechanisms in these clinical circumstances, which is not unexpected given the role of neuroinflammation in neurological diseases (55–57). Consistent with our findings, Silvestro et al. study indicated that an attack following the 2nd dose was considerably more likely to occur if a headache episode had occurred following the 1st dose (23).

#### 4.3.4. Vaccine platform

Headache frequencies reported after SARS-CoV-2 vaccination varied widely between mRNA, adenovirus vector, and inactivated virus (29). This accords with our observations that post-vaccination headaches are more commonly associated with vector vaccines. In corroboration with our findings, a study by Mattiuzzi et al. reported that post-vaccination headaches occurred more frequently among AstraZeneca recipients, followed by Pfizer recipients (35). Similarly, a study among 334 healthcare workers with a history of COVID-19 reported vaccine type as one of the main predictors of post-vaccination headaches, with the highest rate observed for AstraZeneca and Sputnik V (58). Nevertheless, a recent meta-analysis found no significant difference between vaccine types in terms of developing post-vaccination headaches, suggesting these headaches might be secondary to systemic immunological responses than to vaccine-specific reactions (19). It should be noted that more than 90% of individuals in this study received BNT162b2 or ChAdOx1 (19). Generally, with respect to the conflicting results and diverse platforms used in different countries, further investigations and updated meta-analyses might be required to shed light on the effect of different vaccine platforms on post-vaccination headaches.

#### 4.3.5. Fever

Post-vaccination fever was found to be another factor associated with post-vaccination headaches. According to Göbel et al. study, 30.4% of individuals who developed post-vaccination headaches reported fever. Therefore, the authors suggested that inflammatory mediators may play a role in headaches associated with vaccination (30). Our finding also reflects that of Silvestro et al., who indicated a statistically significant relationship between post-vaccination headache attacks and other systemic AEs (23).

### 4.4. Factors associated with developing *prolonged* headaches following SARS-CoV-2 vaccination

To our knowledge, while there are studies evaluating factors associated with prolonged post-COVID-19 headaches (59), no study has been conducted before to specifically investigate the risk factors associated with prolonged headaches following SARS-CoV-2 vaccination. Hence, the literature is still very limited in this field. According to our results, a history of psychiatric disorders may increase the odds of long-lasting headaches following SARS-CoV-2 vaccination. Notably, epidemiological data indicate that unidirectional/bidirectional causal associations between psychiatric disorders and headaches are possible (51). While we found that a history of primary headaches or headaches following COVID-19 might reduce the odds of developing prolonged post-vaccination headaches, Göbel et al. indicated a longer duration of post-vaccination headaches in patients with a history of migraine compared to those without primary headaches (30). According to the authors, the hyperexcitability of trigeminovascular neurons caused by the primary headaches might be attributed to headaches lasting longer following SARS-CoV-2 vaccination (30). However, it can also be hypothesized that people who have had a previous history of headaches may possess better strategies for effectively managing their post-vaccination headaches, potentially leading to the prevention of prolonged headache episodes. More studies are required to enlighten these issues, as well as the effect of other clinicodemographic features (i.e., age, sex, long-lasting headaches following the previous dose, post-vaccination headache type, and vaccine platform) on prolonged post-vaccination headaches.

### 4.5. Limitations and strengths

Our study has several limitations. There were disproportionately more women than men in the sample. Furthermore, although random selection facilitated providing a representative sample of vaccine receivers within the healthcare system and reducing the selection bias, people who did not respond to the invitation might have had lower education or lower socioeconomic status, since the utilization of a web-based questionnaire distributed *via* social media platforms might be less feasible among these groups. This, in turn, may have influenced the reported prevalence of post-vaccination headaches in this study, as individuals with higher education levels may exhibit greater awareness and a higher tendency to report such cases. Additionally, as with previous studies, people with a history of primary headaches or with more severe headaches, as well as those who developed post-vaccination headaches, might have been more willing to engage in this study and, therefore, might be overrepresented. Another possible limitation of the current study, as with other studies, is the possible confounding effect of apprehension about the vaccines’ safety on developing headaches following vaccination.

This study may also be subject to recall bias (responder bias) due to the retrospective recollection retrieved by study participants and the questionnaire-based nature (Recall bias – Catalog of Bias). However, to reduce the recall bias, we tried to define each question and related options clearly to the participant, and the participants also had enough time for adequate recall of long-term memory. Additionally, the questionnaire was designed in chronological events order (history of primary headaches, COVID-19-related headaches, post-1st dose, post-2nd dose, and post-3rd dose headaches). To ensure that no important information from the perspective of the patients was overlooked, open-ended responses were also given and evaluated by an experienced neurologist. To further minimize recall bias, we made an effort to select a reasonable interval (one month) between the last vaccine dose and the distribution of the questionnaires, ensuring it was neither too short nor too long. The strengths of this study include large sample size, a population-based design, the inclusion of different vaccine platforms, and different doses. Of note, since the 3rd dose was taken months apart, the number of events related to this dose was considerably lower. Despite its limitations, this study certainly adds to our understanding of the features and risk factors for post-vaccination headaches and headaches after multiple vaccine doses.

## 5. Conclusions and further directions

Headaches following SARS-CoV-2 vaccination are common adverse events, typically bilateral, moderate, pressing, and responsive to analgesics. They usually occur with a close temporal relationship (10 h) to vaccination and last for nearly 24 h. Factors increasing the risk of post-vaccination headaches include primary headaches, post-COVID-19 headaches, prior vaccine-related headaches, vector-based vaccines, and post-vaccination fever. Primary and post-COVID-19 headaches decrease the likelihood of prolonged post-vaccination headaches, while longer-lasting prior vaccine-related headaches, migraine-like headaches at the same dose, and psychiatric disorders increase the odds of prolonged headaches after vaccination. Understanding the characteristics and risk factors associated with these headaches can help physicians diagnose these headaches and distinguish them from more serious causes (such as CVT) and may also enhance vaccine acceptance and coverage. While studying the factors associated with developing post-vaccination headaches, future studies should take some possible confounding factors (i.e., apprehension about vaccine AEs) into account. Additionally, it is important to note that not all vaccine types may carry the same risk of headaches. Therefore, continued efforts are needed to determine factors associated with headaches and, specifically, *prolonged* headaches following vaccination with various SARS-CoV-2 vaccine platforms. In future studies examining vaccine AEs, it is crucial to consider the vaccine dose as a significant factor, as it has been identified as one of the strongest factors associated with AEs. Lastly, given the high prevalence of headaches attributed to vaccination, continued efforts are needed to update the current ICHD-3 classification system to include vaccines as one of the substances listed in 8.1 Headache attributed to use of or exposure to a substance – ICHD-3.

## Data availability statement

The raw data supporting the conclusions of this article will be made available by the authors upon reasonable request, without undue reservation.

## Ethics statement

This study was approved by the ethics committee of Tehran University of Medical Sciences, Tehran, Iran (IR.TUMS.NI.REC.1400.054). Patients gave their written informed consent for participation and publishing, in accordance with the Declaration of Helsinki.

## Author contributions

MT, EJ, and SN: conception and design. MAL, MJ, and NH: analysis. MJ, MAL, and MYP: interpretation of data. MJ and MYP: drafting. MT, EJ, SN, MAL, and NH: revising. All authors approved the final version to be published and agreed to be accountable for all aspects of the work.

## Conflict of interest

The authors declare that the research was conducted in the absence of any commercial or financial relationships that could be construed as a potential conflict of interest.

## Publisher’s note

All claims expressed in this article are solely those of the authors and do not necessarily represent those of their affiliated organizations, or those of the publisher, the editors and the reviewers. Any product that may be evaluated in this article, or claim that may be made by its manufacturer, is not guaranteed or endorsed by the publisher.

## Footnotes

^1^ https://www.equator-network.org/reporting-guidelines/strobe/

## Abbreviations

CI: Confidence interval
COVID-19: Coronavirus disease 2019
CVT: Cerebral venous thrombosis
V_1_: 1st vaccine dose
V_2_: 2nd vaccine dose
V_3_: 3rd vaccine dose
ICU: Intensive care unit
OR: Odds ratio
P: Probability value
PMH: Past medical history
SARS-CoV-2: Severe acute respiratory syndrome coronavirus 2
