# Supplementary material for: Characteristics of headaches attributed to SARS-CoV-2 vaccination and factors associated with its frequency and prolongation: a cross-sectional cohort study
Source: Front Neurol. 2023 Aug 2;14:1214501. doi: 10.3389/fneur.2023.1214501 (PMC10433229; doi:10.3389/fneur.2023.1214501)
Supplement: Supplementary file 1 [file Data_Sheet_1.PDF]

Supplementary Materials

Table of Contents

Table of Contents		Page No.
Table S1	The Strengthening the Reporting of Observational Studies in Epidemiology (STROBE) Statement	1-5
Table S2	Headache attributed to the infection (ICHD-3, classification 9.)	6-7
Table S3	Headache attributed to the use of or exposure to a substance according to the ICHD-3 classification	8-9
Table S4	COVID-19-related characteristics	10
Table S5	Vaccine-related characteristics	11
Supplementary Result 1	Headache characteristics attributed to COVID-19 infection	12
Table S6	Headaches characteristics attributed to SARS-CoV-2 vaccination for each vaccine exposure	13-14
Supplementary Result 2	Headache characteristics attributed to each dose of SARS-CoV-2 vaccination	15-16
Table S7	Unadjusted impact of variables on developing headaches following SARS-CoV-2 vaccination	17-19
Table S8	Unadjusted impact of variables on developing prolonged headache following SARS-CoV-2 vaccination	20-22

Table S1. STROBE Statement—a checklist of items that should be included in reports of observational studies.

	Item No	Recommendation	Page No
**Title and abstract**	1	(*a*) Indicate the study's design with a commonly used term in the title or the abstract	1
		(*b*) Provide in the abstract an informative and balanced summary of what was done and what was found	1
**Introduction**			
Background/rationale	2	Explain the scientific background and rationale for the investigation being reported	1
Objectives	3	State specific objectives, including any prespecified hypotheses	2
**Methods**			
Study design	4	Present key elements of study design early in the paper	3
Setting	5	Describe the setting, locations, and relevant dates, including periods of recruitment, exposure, follow-up, and data collection	3,4
Participants	6	(*a*) *Cohort study*—Give the eligibility criteria and the sources and methods of selection of participants. Describe methods of follow-up<br>*Case-control study*—Give the eligibility criteria and the sources and methods of case ascertainment and control selection. Give the rationale for the choice of cases and controls.<br>*Cross-sectional study*—Give the eligibility criteria and the sources and methods of selection of participants	3, 4

		(*b*) *Cohort study*—For matched studies, give matching criteria and the number of exposed and unexposed<br>*Case-control study*—For matched studies, give matching criteria and the number of controls per case	
Variables	7	Clearly define all outcomes, exposures, predictors, potential confounders, and effect modifiers. Give diagnostic criteria, if applicable	4-6
Data sources/ measurement	8*	For each variable of interest, give sources of data and details of methods of assessment (measurement). Describe the comparability of assessment methods if there is more than one group	4-6
Bias	9	Describe any efforts to address potential sources of bias	20
Study size	10	Explain how the study size was arrived at	3
Quantitative variables	11	Explain how quantitative variables were handled in the analyses. If applicable, describe which groupings were chosen and why	7
Statistical methods	12	(*a*) Describe all statistical methods, including those used to control for confounding	7
		(*b*) Describe any methods used to examine subgroups and interactions	NA
		(*c*) Explain how missing data were addressed	7
		(*d*) *Cohort study*—If applicable, explain how the loss to follow-up was addressed<br>*Case-control study*—If applicable, explain how the matching of cases and controls was addressed	NA

	Cross-sectional study—If applicable, describe analytical methods taking account of the sampling strategy	
	(*e*) Describe any sensitivity analyses	NA

Continued on the next page

Results

Participants	13*	(a) Report numbers of individuals at each stage of study—e.g., numbers potentially eligible, examined for eligibility, confirmed eligible, included in the study, completing follow-up, and analyzed	8
		(b) Give reasons for non-participation at each stage	8
		(c) Consider the use of a flow diagram	Figure 1
Descriptive data	14*	(a) Give characteristics of study participants (e.g., demographic, clinical, social) and information on exposures and potential confounders	8
		(b) Indicate the number of participants with missing data for each variable of interest	NA
		(c) *Cohort study*—Summarise follow-up time (e.g., average and total amount)	NA
Outcome data	15*	*Cohort study*—Report numbers of outcome events or summary measures over time	NA
		Case-control study—Report numbers in each exposure category or summary measures of exposure	NA
		Cross-sectional study—Report numbers of outcome events or summary measures	9-10
Main results	16	(*a*) Give unadjusted estimates and, if applicable, confounder-adjusted estimates and their precision (e.g., 95% confidence interval). Make clear which confounders were adjusted for and why they were included	10-13
		(*b*) Report category boundaries when continuous variables were categorized	12

		(*c*) If relevant, consider translating estimates of relative risk into absolute risk for a meaningful time period	NA
Other analyses	17	Report other analyses are done—e.g., analyses of subgroups and interactions and sensitivity analyses.	NA
**Discussion**			
Key results	18	Summarise key results with reference to study objectives	14
Limitations	19	Discuss the limitations of the study, taking into account sources of potential bias or imprecision. Discuss both the direction and magnitude of any potential bias	19
Interpretation	20	Give a cautious overall interpretation of results considering objectives, limitations, the multiplicity of analyses, results from similar studies, and other relevant evidence	15-19
Generalisability	21	Discuss the generalisability (external validity) of the study results	19-20
**Other information**			
Funding	22	Give the source of funding and the role of the funders for the present study and, if applicable, for the original study on which the present article is based	23

Note: **STROBE Statement—a checklist of items that should be included in reports of observational studies.**
*Give information separately for cases and controls in case-control studies and, if applicable, for exposed and unexposed groups in the cohort and cross-sectional studies.

Table S2. Headache attributed to the infection (ICHD-3, classification 9.)

ICHD-3	Headache	Description	Diagnostic Criteria	Comments
9.	**Headache attributed to infection**		**A.** Headache fulfilling criterion C.<br>**B.** An infection, or sequela of infection, known to be able to cause headaches has been diagnosed.<br>**C.** Evidence of causation demonstrated by at least two of the following:<br>1. headache has developed in temporal relation to the onset of the infection.<br>2. either or both of the following:<br>a) headache has significantly worsened in parallel with the worsening of the infection.<br>b) headache has significantly improved or resolved in parallel with the improvement in or resolution of the infection.<br>3. headache has characteristics typical for the infection.<br>**D.** Not better accounted for by another ICHD-3 diagnosis.	• The triad of headache, fever, and nausea/vomiting is highly suggestive of 9. Headache attributed to infection. The probability is increased when lethargy or convulsions are also part of the clinical picture.<br>• The general rules for attribution to another disorder apply to 9. Headache attributed to infection.<br>• When a new headache occurs for the first time in close temporal relation to an infection, it is coded as a secondary headache attributed to that infection. This remains true when the new headache has the characteristics of any of the primary headache disorders classified in Part One of ICHD-3.<br>• When a pre-existing headache with the characteristics of a primary headache disorder becomes chronic or is made significantly worse (usually meaning a two-fold or greater increase in frequency and/or severity), in close temporal relation to infection, both the initial headache diagnosis and a diagnosis of 9. Headache attributed to infection (or one of its types or subtypes) should be given, provided that there is good evidence that the disorder can cause headaches.<br>• Headache attributed to infection is usually the consequence of active infection, resolving within 3 months of eradication of the infection. In some cases, depending on the pathogenic agent, the infection cannot be treated effectively and remains active. The headache in these cases may not abate because the cause remains present; after 3 months, both the infection and the headache are referred to as chronic.<br>• In other, rarer cases, the infection resolves or is eradicated, but the headache does not remit; after 3 months, such a headache is termed persistent (in keeping with other secondary headaches).<br>• Accordingly, acute and chronic subforms of headache attributed to active or recent infection have been defined, in some cases, in contrast to persistent subforms of post-infectious headache (see, for example, 9.1.1.1 Acute headache attributed to bacterial meningitis or meningoencephalitis, 9.1.1.2 Chronic headache attributed to bacterial meningitis or meningoencephalitis and 9.1.1.3 Persistent headache attributed to past bacterial meningitis or meningoencephalitis). The purpose is to distinguish and keep separate two probably different causative mechanisms and two different management approaches.
9.2	**Headache attributed to systemic infection**	Headache of variable duration caused by systemic infection, usually accompanied by other symptoms and/or clinical signs of the infection.	-	• Headache in systemic infections is usually a relatively inconspicuous symptom and diagnostically unhelpful. These conditions are mostly dominated by fever, general malaise, and other systemic symptoms.<br>• Nevertheless, some systemic infections, particularly influenza, have headaches as a prominent symptom along with fever and others. When systemic infection is accompanied by meningitis or encephalitis, any headache attributed to the infection should be coded to these disorders as a subtype or subform of 9.1 Headache attributed to intracranial infection.<br>• In infectious diseases, headache commonly coexists with fever and may be dependent on it, but headache can also occur in the absence of fever. The exact nature of these mechanisms remains to be investigated. Meanwhile, the great variability in their

				propensity for causing headaches indicates that systemic infections do not have this effect simply through fever and exogenous or endogenous pyrogens.<br>• The mechanisms causing headaches include the direct effects of the microorganisms themselves. Several cells are likely to be involved (activated microglia and monocytic macrophages, activated astrocytes and blood-brain barrier and endothelial cells), along with several immunoinflammatory mediators (cytokines, glutamate, COX-2/PGE2 system, NO–iNOS system and reactive oxygen species system).
**9.2.2**	**Headache attributed to systemic viral infection**	Headache caused by and occurring in association with other symptoms and/or clinical signs of a systemic viral infection in the absence of meningitis or encephalitis.	**A.** Headache of any duration fulfilling criterion C<br>**B.** Both of the following:<br>1. systemic viral infection has been diagnosed.<br>2. no evidence of meningitic or encephalitic involvement<br>**C.** Evidence of causation demonstrated by at least two of the following:<br>1. headache has developed in temporal relation to the onset of the systemic viral infection.<br>2. headache has significantly worsened in parallel with the worsening of the systemic viral infection.<br>3. headache has significantly improved or resolved in parallel with the improvement in or resolution of the systemic viral infection.<br>4. headache has either or both of the following characteristics:<br>a) diffuse pain<br>b) moderate or severe intensity<br>**D.** Not better accounted for by another ICHD-3 diagnosis.	-

Note: References**:** 9. Headache attributed to infection - ICHD-3; 9.2 Headache attributed to systemic infection - ICHD-3; 9.2.2 Headache attributed to systemic viral infection - ICHD-3.

Table S3. Headache attributed to the use of or exposure to a substance according to the ICHD-3 classification (ICHD-3, classification 8.1)

ICHD-3	Headache	Description	Diagnostic Criteria	Comments
**8.1**	**Headache attributed to the use of or exposure to a substance**	Headache is caused by the use of or exposure to a substance, with onset immediately or within hours.	-	• Headache attributed to the use of or exposure to a substance can be an unwanted effect of a substance in normal therapeutic use or in experimental studies or caused by a toxic substance.<br>• Headache as a side-effect has been recorded with many drugs, often merely reflecting the high prevalence of headaches. Only when it occurs more often after an active drug than after a placebo in double-blind controlled trials can headache be regarded as a true side-effect.<br>• The double-blind design can also be used experimentally to study the relationship between drug effects and headaches. In some cases, for example, nitric oxide (NO) donors, such studies have led to a deeper understanding of the involvement of neurotransmitter mechanisms in primary headaches.<br>• In general, people with 1. Migraine are much more susceptible to such headaches than other individuals, and the same may be true for people with 2. Tension-type headache or 3.1 Cluster headache.<br>• Several substances, such as NO donors and histamine, induce an immediate headache in both normal volunteers and migraineurs. However, it is now clear that people who have primary headache disorders may also develop a delayed headache, one to several hours after the substance has been cleared from the blood.<br>• Knowledge of the potential headache-inducing effects of substances in clinical use is important to label these substances appropriately.<br>• Combinations such as alcohol and disulfiram may cause headaches when individual agents might not.<br>• Paradoxically, the headache encountered by most people after heavy alcohol intake may be a positive feature because it encourages the avoidance of excessive drinking.<br>• Substances that cause headaches through their toxic effects, such as carbon monoxide, cannot be studied experimentally. The causal relationship between exposure and headache has, therefore, should be demonstrated in clinical cases where the substance has been used accidentally or for a suicide attempt.

**8.1.9**	**Headache attributed to the occasional use of non-headache medication**	Headache occurs as an acute adverse event after occasional use of medication taken for purposes other than the treatment of headache.	**A.** Any headache fulfilling criterion C.<br>**B.** One or more doses of medication have been taken for purposes other than the treatment of headache.<br>**C.** Evidence of causation is demonstrated by both of the following:<br>1. headache has developed within minutes to hours of intake.<br>2. headache has resolved within 72 hours after intake has ceased.<br>**D.** Not better accounted for by another ICHD-3 diagnosis.	• Headache attributed to the occasional use of non-headache medication has been reported as an adverse event after the use of many drugs.<br>• The following are the most incriminated: atropine, digitalis, disulfiram, hydralazine, imipramine, nicotine, nifedipine, nimodipine, and sildenafil.<br>• The headache characteristics are not very well defined in the literature and probably depend on the drug, but in most cases, headache is dull, continuous, diffuse, and of moderate to severe intensity.
**8.1.11**	**Headache attributed to the use of or exposure to other substances**	Headache occurring during or soon after, and caused by, use of or exposure to a substance other than those described above, including herbal, animal, or other organic or inorganic substances given by physicians or non-physicians with medicinal intent although not licensed as medicinal products.	**A.** Any headache fulfilling the criterion C.<br>**B.** Exposure has occurred to a substance other than those described above.<br>**C.** Evidence of causation is demonstrated by both of the following:<br>1. headache has developed within 12 hours of exposure.<br>2. headache has resolved within 72 hours after exposure.<br>**D.** Not better accounted for by another ICHD-3 diagnosis.	• Headache attributed to the use of or exposure to other substances includes headache caused by the herbal, animal, or other organic or inorganic substances given by physicians or non-physicians with medicinal intent, although not licensed as medicinal products.<br>• It has been reported after exposure to several other organic and inorganic substances.<br>• Inorganic compounds: arsenic, borate, bromate, chlorate, copper, iodine, lead, lithium, mercury, and tolazoline hydrochloride.<br>• Organic compounds: aniline, balsam, camphor, carbon disulfide, carbon tetrachloride, chlordecone, EDTA, heptachlor, hydrogen sulfide, kerosene, long-chain alcohols, methyl alcohol, methyl bromide, methyl chloride, methyl iodine, naphthalene, organophosphorus compounds (parathion, pyrethrum).<br>• The characteristics of 8.1.11 Headache attributed to the use of or exposure to other substances are not well defined in the literature and almost certainly vary with the agent. In most cases, it is dull, diffuse, continuous, and of moderate to severe intensity.

Note: References: 8.1 Headache attributed to use of or exposure to a substance - ICHD-3; 8.1.9 Headache attributed to occasional use of non-headache medication - ICHD-3; 8.1.11 Headache attributed to use of or exposure to other substance - ICHD-3.

Table S4. COVID-19-related characteristics

Variable		Frequency (%)
**History of COVID-19**		780 (42.8)
**COVID-19 severity**	Home quarantine	732 (93.8)
	Ward admission	43 (5.5)
	ICU admission	5 (0.6)
**COVID-19 manifestation**	Respiratory	373 (47.8)
	Systemic	216 (27.7)
	Gastrointestinal	129 (16.5)
	Neurological§	62 (7.7)

Note: Categorial data are presented as numbers (%). Abbreviations: COVID-19: coronavirus disease 2019; ICU: intensive care unit.

§ Manifesting with neurological symptoms, not including headache (vertigo, olfactory dysfunction, seizures, altered mental status, stroke, etc.)

Table S5. Vaccine-related characteristics

Variable		1st dose (N= 1822)	2nd dose (N= 1122)	3rd dose (N= 203)	Total (N = 3147)
Vaccine type	Sinopharm	634 (34.8)	524 (46.7)	55 (27.1)	1213 (38.5)
	AstraZeneca	530 (29.1)	208 (18.5)	98 (48.3)	836 (26.6)
	Sputnik-v	503 (27.6)	290 (25.8)	2 (1.0)	795 (25.3)
	Pfizer–BioNTech	46 (2.5)	40 (3.6)	13 (6.4)	99 (3.1)
	Baharat	54 (3.0)	28 (2.5)	0	82 (2.6)
	Barekat	26 (1.4)	27 (2.4)	3 (1.5)	56 (1.8)
	PastoCovac	0	0	14 (6.9)	14 (0.4)
	Spikogen	0	0	8 (3.9)	8 (0.3)
	Razi	2 (0.1)	2 (0.2)	4 (2.0)	8 (0.3)
	Moderna	2 (0.1)	0	0	2 (0.1)
	Noora	0	0	1 (0.5)	1 (0.03)
	Fakhra	0	0	1 (0.5)	1 (0.03)
**Analgesics use before vaccination †**		217 (11.9)	125 (11.1)	16 (7.9)	358 (11.4)
**Nonserious complications ‡**		1263 (69.3)	693 (61.8)	135 (66.5)	2091 (66.4)
**Post-vaccination fever**		628 (34.5)	224 (20.0)	66 (32.5)	918 (29.2)
**COVID-19 infection after vaccination§**		54 (3.0)	17 (1.5)	11 (5.4)	82 (2.6)

Note: Categorial data are presented as numbers (%). Abbreviations: COVID-19: coronavirus disease 2019.
† Using analgesics, such as acetaminophen, naproxen, ibuprofen, etc., before receiving the vaccine.
‡ Nonserious complications of vaccines were defined as local injection-site reactions (pain, skin irritation, swelling, erythema), weakness/ fatigue, fever< 40, chill, anorexia, dizziness, vertigo, syncope, myalgia, headaches, arthralgia, sore throat, nasal congestion or rhinorrhea, nausea/ vomiting, and diarrhea.
§ Defined as a confirmed diagnosis of COVID-19 at least 7 days after receiving the vaccine.

Supplementary Results 1. Headache characteristics attributed to COVID-19 infection

Of 780 individuals with a history of COVID-19, 287 (36.8%) reported headaches following COVID-19. Of 287 patients with headaches following COVID-19 infection, 178 (62.0%) had a history of primary headaches, of whom 74 (41.6%) declared experiencing similar post-COVID-19 headaches to their primary headaches. Post-COVID-19 headaches were migraine-like and tension-type in 59.2% and 31.4%, respectively. The most common headache qualities were described as pressing (33.8%) and dull aches (29.3%). Most patients reported moderate (49.8%) and bilateral (70.4%) headaches, affecting multiple head/neck regions or the entire head (55.8%). The median duration between COVID-19 manifestation and headaches onset was 24.0 hours [2.0, 24.0], with headaches initiated in less than two days from COVID-19 onset in 66.9% of the individuals. Each headache attack lasted three hours [1.0, 5.0], with the majority of patients experiencing attack durations of less than 24 hours (55.7%). The headache was most severe 2 days after the infection [2.0, 4.0] and lasted for a median of 5 days [2.0, 10.0]. Post-COVID-19 headaches lasted for more than 48 hours in nearly 80% of patients.

Table S6. Headaches characteristics attributed to SARS-CoV-2 vaccination for each vaccine exposure

Variable		1st dose (N= 665)	2nd dose (N= 261)	3rd dose (N= 44)
**Primary headaches history**		381 (57.3)	165 (63.2)	27 (61.4)
**Primary headaches type**	Migraine	165 (43.3)	66 (40.0)	13 (48.1)
	Tension-type	148 (38.8)	65 (39.4)	10 (37.0)
	Cluster	11 (2.9)	10 (6.1)	1 (3.7)
	Other	57 (15.0)	24 (14.5)	3 (11.1)
**Similarity to primary headaches§**		255/381 (66.9)	62/165 (37.6)	15/27 (55.6)
**History of COVID-19 headaches**		115 (17.3)	60 (23.0)	21 (47.7)
**Similarity to COVID-19 headaches§**		81/115 (70.4)	40/60 (66.7)	16/21 (76.2)
**History of post-1st dose headaches**		-	147 (56.3)	19 (43.2)
**Similarity to post-1st dose headaches§**		-	95/147 (64.6)	12/19 (63.2)
**History of post-2nd dose headaches**		-	-	17 (38.6)
**Similarity to post-2nd dose headaches§**		-	-	11/17 (64.7)
**Headaches type**	Migraine-like	225 (33.8)	96 (36.8)	29 (65.9)
	Tension-type	317 (47.7)	122 (46.7)	12 (27.3)
	Undifferentiated	123 (18.5)	43 (16.5)	3 (6.8)
**Headaches intensity**	Mild	121 (18.2)	59 (22.6)	7 (15.9)
	Moderate	354 (53.2)	122 (46.7)	19 (43.2)
	Severe	190 (28.6)	80 (30.7)	18 (40.9)
**Headaches quality**	Dull ache	91 (13.7)	30 (11.5)	8 (18.2)
	Sharp	98 (14.7)	46 (17.6)	9 (20.5)
	Pressing	370 (55.6)	139 (53.3)	18 (40.9)
	Pulsatile/throbbing	100 (15.0)	39 (14.9)	8 (18.2)
**Headaches location**	Frontal	109 (16.4)	31 (11.9)	4 (9.1)
	Temporal	87 (13.1)	27 (10.3)	7 (15.9)
	Occipital	27 (4.1)	13 (5.0)	0
	Top of the head	48 (7.2)	12 (4.6)	0
	Entire head	169 (25.4)	88 (33.7)	17 (38.6)
	Nuchal region‖	2 (0.3)	1 (0.4)	0
	Entire head and nuchal region‖	22 (3.3)	16 (6.1)	4 (9.1)
	Multiple†	193 (29.0)	65 (24.9)	12 (27.3)
**Headaches lateralization**	Unilateral/ more prominent on one side	204 (30.7)	79 (30.3)	11 (25.0)
	Bilateral	461 (69.3)	182 (69.7)	33 (75.0)
**Time to onset (h)**		10.0 [5.0, 24.0]	8.0 [4.0, 24.0]	10.0 [5.0, 12.0]
**Time to onset, categorical**	<1 d	468 (70.4)	187 (71.6)	35 (79.5)

	1≤onset<2 d	100 (15.0)	32 (12.3)	5 (11.4)
	2≤onset<7 d	55 (8.3)	20 (7.7)	3 (6.8)
	≥7 d	21 (3.2)	14 (5.4)	0
**Time to maximum intensity (d/h)**		10 h [2.0, 24.0]	24.0 h [16.0, 48.0]	24.0 h [24.0, 48.0]
**Attack duration (h)**		2.0 [2.0, 3.0]	NA	3.0 [1.0, 6.0]
**Attack duration, categorical**	<1 h	12 (1.8)	31 (11.9)	1 (2.3)
	1≤duration<4 h	438 (65.9)	99 (37.9)	16 (36.4)
	4≤duration<24 h	113 (17.0)	62 (23.8)	12 (27.3)
	24≤duration<72 h	11(1.7)	15 (5.7)	0
	≥72 h	18 (2.7)	19 (7.3)	4 (9.1)
**Headaches end (d/h)**		12.0 h [2.0, 48.0]	48.0 h [24.0, 72.0]	48.0 h [24.0, 72.0]
**Headaches end, categorical**	<48 h	419 (63.0)	117 (44.8)	19 (43.2)
	≥48 h	203 (30.5)	124 (47.5)	23 (52.3)
**Analgesic use**‡		562 (84.5%)	223 (85.4)	39 (88.6)
**Analgesic response**	<50%	187 (33.3)	78 (35.0)	17 (43.6)
	≥50%	357 (63.5)	140 (62.8)	22 (56.4)

Note: Categorial data are presented as numbers (%). Asymmetric numeric data are described using median [IQR]. Abbreviations: IQR: interquartile range, COVID-19: coronavirus disease 2019, SARS-CoV-2: severe acute respiratory syndrome coronavirus 2, d: day(s), h: hour(s).

† At least two locations at the head or neck.

‡ Using analgesics, such as acetaminophen, naproxen, ibuprofen, etc. for headaches.

§ A general inquiry was used to subjectively assess the similarity between the patients' post-vaccination headaches and their prior headaches, based on their own opinion, without specifically focusing on any particular characteristics.

|| Nuchal region was defined as the posterior part of the upper neck, which includes the region where the neck muscles attach to the cranium.

Supplementary Results 2. Headache characteristics attributed to each dose of SARS-CoV-2 vaccination

Supplementary Table S6 shows headache characteristics following the 1st, 2nd, and 3rd SARS-CoV-2 vaccine dose.

1st dose: Of 1822 1st-dose receivers, 665 (36.5%) reported headaches, of whom 57.3% and 17.3% had a history of primary headaches and COVID-19 headaches, respectively. Similarities between post-1st dose headaches with the primary headaches and post-COVID-19 headaches were reported by 66.9% and 70.4% of the individuals, respectively. Migraine-like and TTH were found in 33.8% and 47.7% of participants, respectively. Post-1st dose headaches were mostly bilateral (69.3%) with moderate intensity (53.2%) and pressing quality (55.6%). Headaches mostly affected multiple regions of the head/neck (29.0%) or the entire head (25.4%). Headache onset was less than 2 days after vaccination in most of the participants (85.4%), with a median of 10 hours after vaccination [5.0, 24.0] and the majority (70.4%) initiated in less than a day. Post-1st dose headaches reached their maximum intensity 10 hours after vaccination [2.0, 24.0]. The median attack duration was 2 hours [2.0, 3.0], with most of the patients (65.9%) reporting a duration between one to four hours. Less than five percent of the participants experienced attack durations of more than 24 hours. The headaches ended 12 hours after vaccination [2.0, 48.0], ranging from a half-hour to 21 days, with the majority ending in less than 48 hours (63.0%). Of 84.5% of patients who used analgesics for their headaches, 63.5% responded more than 50%.

2nd dose: Of 1122 2nd-dose receivers, 261 (23.3%) reported headaches, of whom 46.7% and 36.8% had TTH and migraine-like headaches, respectively. A history of primary headaches was reported by 63.2%, of whom 37.6% reported similar post-2nd dose headaches to their primary headaches. Post-COVID-19 headache was reported by 23.0%, of whom 66.7% reported similar post-2nd dose headaches. Notably, 56.3% of individuals with post-2nd dose headaches also had post-1st dose headaches, of whom 64.6% reported similar headaches. Post-2nd dose headaches were mostly bilateral (69.7%), with moderated intensity (46.7%) and pressing quality (53.3%). Headaches were initiated less than a day after vaccination in most

of the patients (71.6%), with a median of 8 hours [4.0, 24.0]. They ended 48 hours later [24.0, 72.0], ranging from 4 hours to 30 days. The attack duration was less than 24 hours in most of the individuals (73.6%). However, 13.0% experienced attack durations of more than 24 hours. Patients reported experiencing the maximum intensity of headaches 24 hours following vaccination [16.0, 48,0]. Of 85.4% who used analgesics, 62.8% reported more than 50% response.

3rd dose: Among 203 3rd-dose receivers, 44 (21.7%) reported headaches, of whom 61.4%, 47.7%, 43.2%, and 38.6% had a history of primary headaches, COVID-19-related headaches, post-1st dose headaches, and post-2nd dose headaches, respectively. Of note, 55.6% reported similar post-3rd headaches to their primary headaches. Additionally, 76.2%, 63.2%, and 64.7% reported similar post-3rd dose headaches to their COVID-19 headaches, post-1st dose headaches, and post-2nd dose headaches, respectively. Post-3rd dose migraine-like and TTH were found in 65.9% and 27.3% of individuals, respectively. Most of the patients reported moderate (43.2%) and bilateral (75.0%) headaches, with a pressing quality (40.9%). Headaches were initiated 10 hours [5.0, 12.0] after the 3rd dose, with the majority of participants (79.5%) reporting headache onset less than a day following vaccination. The attack duration was 3.0 hours [1.0, 6.0]. The headaches reached their maximum intensity 24 hours [24.0, 48.0] after vaccination and lasted for a median of 48.0 hours [24.0, 72.0], ranging from 24 hours to 14 days. Of 88.6% of patients who used analgesics, more than half (56.4%) responded.

Table S7. Unadjusted impact of variables on developing headaches following SARS-CoV-2 vaccination

Variables	1st dose		2nd dose		3rd dose	
	OR (95% CI)	P	OR (95% CI)	P	OR (95% CI)	P
**Age**	1.02 (1.01, 1.02)	**0.001****	1.03 (1.01, 1.05)	**<0.001*****	1.01 (0.98, 1.05)	0.414
**Sex (F vs. M)**	1.82 (1.45, 2.27)	**<0.001*****	1.67 (1.19, 2.33)	**0.003****	1.64 (0.82, 3.45)	0.169
**PMH**						
Thyroid disorders	1.06 (0.67, 1.68)	0.803	1.20 (0.60, 2.38)	0.609	0.21 (0.14, 0.32)	**<0.001*****
Psychiatric disorders†	1.51 (0.87, 2.63)	0.146	0.71 (0.24, 2.06)	0.529	3.11 (0.75, 11.66)	0.095
HTN	1.70 (0.97, 2.98)	0.064	2.10 (0.84, 5.20)	0.111	2.33 (0.31, 12.60)	0.342
Autoimmune disorders	0.71 (0.39, 1.30)	0.268	0.83 (0.28, 2.45)	0.740	3.50 (0.66, 16.77)	0.114
Respiratory disorders	0.78 (0.42, 1.47)	0.445	1.60 (0.70, 3.64)	0.266	4.67 (0.83, 26.42)	0.068
Cardiovascular disorders‡	1.01 (0.45, 2.26)	0.987	1.44 (0.39, 5.30)	0.586	1.33 (0.19, 5.89)	0.729
DM	1.81 (0.55, 5.97)	0.328	7.18 (1.19, 43.44)	**0.032***	4.67 (0.18, 120.50)	0.281
**Primary headaches history**	1.40 (1.16, 1.70)	**<0.001*****	1.49 (1.12, 1.98)	**0.006****	1.61 (0.82, 3.23)	0.172
**Primary headaches type**						
Migraine vs. Tension-type	0.88 (0.65, 1.17)	0.376	0.71 (0.47, 1.07)	0.099	1.82 (0.69, 4.081)	0.227
Cluster vs. Tension-type	0.77 (0.35, 1.69)	0.516	1.27 (0.55, 2.95)	0.580	1.75 (0.14, 21.35)	0.661
**History of COVID-19 infection**	1.24 (1.02, 1.50)	**0.028***	0.77 (0.58, 1.02)	0.065	1.64 (0.82, 3.41)	0.169
**COVID-19 severity**						
Ward admission vs. home quarantine	1.23 (0.66, 2.28)	0.517	4.63 (1.74, 12.30)	**0.002****	1.54 (0.07, 16.62)	0.730
ICU admission vs. home quarantine	0.39 (0.04, 3.49)	0.398	2.06 (0.19, 22.92)	0.558	1.54 (0.07, 16.62)	0.730
**COVID-19 manifestation**						
Systemic vs. Respiratory	0.94 (0.66, 1.32)	0.705	1.27 (0.78, 2.07)	0.344	0.90 (0.31, 2.44)	0.843
Gastrointestinal vs. Respiratory	1.18 (0.79, 1.78)	0.420	0.90 (0.47, 1.72)	0.751	0.72 (0.19, 2.33)	0.605
Neurological vs. Respiratory	1.31 (0.76, 2.25)	0.329	1.11 (0.50, 2.49)	0.792	0.68 (0.10, 3.04)	0.642
**History of COVID-19 headaches**	1.05 (0.78, 1.41)	0.757	1.43 (0.93, 2.19)	0.103	2.55 (1.08, 6.43)	**0.038***
**COVID-19 headaches type**						
Migraine-like vs. Tension-type	1.33 (0.79, 2.26)	0.288	4.56 (1.03, 20.29)	**0.046***	3.80 (1.15, 15.15)	**0.038***
Undifferentiated vs. Tension-type	1.25 (0.52, 3.01)	0.624	4.27 (0.94, 19.40)	0.060	1.19 (0.05, 11.30)	0.890
**COVID-19 headaches intensity**						
Moderate vs. Mild	1.46 (0.62, 3.41)	0.385	1.34 (0.70, 2.57)	0.375	1.17 (0.22, 9.03)	0.860
Severe vs. Mild	1.84 (0.77, 4.37)	0.170	1.17 (0.41, 3.35)	0.777	2.14 (0.39, 16.75)	0.405
**COVID-19 headaches time to onset**						
<1 d vs. Manifesting	1.19 (0.58, 2.48)	0.635	0.97 (0.41, 2.30)	0.939	2.10 (0.46, 10.41)	0.346
1≤onset<2 d vs. Manifesting	0.69 (0.33, 1.45)	0.333	0.48 (0.19, 1.23)	0.127	0.37 (0.07, 1.82)	0.225
2≤onset<7 d vs. Manifesting	0.61 (0.27, 1.38)	0.237	1.17 (0.47, 2.91)	0.737	0.16 (0.01, 1.26)	0.123
≥7 d vs. Manifesting	1.23 (0.07, 20.76)	0.887	2.42 (0.14, 41.87)	0.544		

**COVID-19 headaches attack duration**	1.00 (0.98, 1.02)	0.892	1.03 (1.00, 1.06)	**0.023***	1.01 (0.97, 1.05)	0.604
**COVID-19 headaches attack duration, categorical**						
1≤duration<4 h vs. <1 h	1.14 (0.35, 3.73)	0.830	3.10 (0.38, 25.47)	0.293	1.07 (0.09, 25.05)	0.960
4≤duration<24 h vs. <1 h	1.47 (0.42, 5.16)	0.551	4.71 (0.55, 40.71)	0.159	0.75 (0.05, 19.73)	0.837
24≤duration<72 h vs. <1 h	1.02 (0.24, 4.41)	0.981	5.00 (0.50, 50.07)	0.171	1.33 (0.07, 41.63)	0.851
≥72 h vs. <1 h hour	1.26 (0.34, 4.67)	0.726	3.50 (0.38, 32.12)	0.268	1.00 (0.06, 26.86)	1.000
**COVID-19 headaches end**	1.03 (1.01, 1.06)	**0.011***	1.01 (0.98, 1.03)	0.608	1.03 (0.94, 1.12)	0.571
**COVID-19 headache end, categorical**						
≥48 h vs. <48 h	2.69 (1.05, 6.92)	**0.040***	3.46 (0.78, 15.32)	0.101	2.57 (0.38, 51.06)	0.405
**History of post-1st dose headaches**	-	-	7.81 (5.73, 10.66)	**<0.001*****	3.72 (1.79, 7.71)	**<0.001*****
**Post-1st dose headaches type**						
Migraine-like vs. Tension-type	-	-	1.81 (1.07, 3.07)	**0.028***	3.14 (0.90, 11.77)	0.078
Undifferentiated vs. Tension-type	-	-	0.50 (0.23, 1.08)	0.077	0.76 (0.03, 7.24)	0.826
**Post-1st dose headaches intensity**						
Moderate vs. Mild	-	-	1.02 (0.49, 2.18)	0.958	1.00 (0.19, 5.83)	1.000
Severe vs. Mild	-	-	2.87 (1.24, 6.87)	**0.015***	1.43 (0.24, 9.49)	0.698
**Post-1st dose headaches intensity**						
Severe vs. Non-severe	-	-	2.83 (1.49, 5.55)	**0.002****	1.43 (0.38, 5.33)	0.592
**Post-1st dose headaches time to onset**						
1≤onset<2 d vs. <1 d	-	-	0.86 (0.43, 1.75)	0.676	0.75 (0.09, 4.40)	0.758
2≤onset<7 d vs. <1 d	-	-	2.04 (0.43, 14.47)	0.401	1.50 (0.06, 40.08)	0.781
≥7 d vs. <1 d	-	-	4.89 (0.82, 93.31)	0.145	1.01 (0.93, 1.09)	0.782
**Post-1st dose headaches attack duration**	-	-	1.01 (0.96, 1.07)	0.805	NA	NA
**Post-1st dose headaches attack duration, categorical**						
1≤duration<4 h vs. <1 h	-	-	4.20 (1.15, 19.90)	**0.041***	NA	NA
4≤duration<24 h vs. <1 h	-	-	5.48 (1.40, 27.38)	**0.021***	NA	NA
24≤duration<72 h vs. <1 h	-	-	2.00 (0.26, 16.04)	0.497	NA	NA
≥72 h vs. <1 h hour	-	-	3.05 (0.61, 18.44)	0.191	NA	NA
**Post-1st dose headaches end**	-	-	1.01 (1.00, 1.02)	0.122	1.08 (0.87, 1.35)	0.475
**Post-1st dose headaches end, categorical**						
≥48 h vs. <48 h	-	-	2.71 (1.21, 6.69)	**0.020***	NA	NA
**History of post-2nd dose headaches**	-	-	-	-	11.18 (4.51, 29.90)	**<0.001*****
**Post-2nd dose headaches type**						
Migraine-like vs. Tension-type	-	-	-	-	1.29 (0.23, 7.35)	0.772
Undifferentiated vs. Tension-type	-	-	-	-	NA	NA
**Post-2nd dose headaches intensity**						
Moderate vs. Mild	-	-	-	-	2.33 (0.28, 21.10)	0.428
Severe vs. Mild	-	-	-	-	2.50 (0.26, 29.56)	0.433

**Post-2nd dose headaches intensity**						
Severe vs. Non-severe	-	-	-	-	1.50 (0.23, 12.86)	0.680
**Post-2nd dose headaches time to onset**						
1≤onset<2 d vs. <1 d	-	-	-	-	NA	NA
2≤onset<7 d vs. <1 d	-	-	-	-	NA	NA
≥7 d vs. <1 d	-	-	-	-	NA	NA
**Post-2nd dose headaches attack duration**						
1≤duration<4 h vs. <1 h	-	-	-	-	NA	NA
4≤duration<24 h vs. <1 h	-	-	-	-	NA	NA
24≤duration<72 h vs. <1 h	-	-	-	-	NA	NA
≥72 h vs. <1 h hour	-	-	-	-	NA	NA
**Post-2nd dose headaches end**	-	-	-	-	1.00 (0.98, 1.02)	0.992
**Post-2nd dose headaches end, categorical**						
≥48 h vs. <48 h	-	-	-	-	1.80 (0.27, 11.53)	0.528
**Vaccine platform[§]**						
Vector vs. Inactivated	2.80 (2.27, 3.46)	**<0.001*** **	3.11 (2.32, 4.21)	**<0.001*** **	4.43 (1.84, 12.44)	**0.002** **
Protein Subunit vs. Inactivated	3.23 (0.20, 51.84)	0.409	6.06 (0.24, 154.28)	0.204	1.64 (0.39, 6.30)	0.477
mRNA vs. Inactivated	0.85 (0.41, 1.74)	0.654	1.52 (0.63, 3.25)	0.314	0.00 (0.00,	0.989
**Analgesics use before vaccination[‖]**	0.61 (0.46, 0.81)	**<0.001*** **	2.48 (1.68, 3.65)	**<0.001*** **	1.23 (0.33, 3.74)	0.737
**Post-vaccination fever[¶]**	5.41 (4.39, 6.68)	**<0.001*** **	5.03 (3.67, 6.90)	**<0.001*** **	9.80 (4.68, 21.77)	**<0.001*** **

Note: Abbreviations: OR: odds ratio, CI: confidence interval, P: probability value, COVID-19: coronavirus disease 2019, SARS-CoV-2: severe acute respiratory syndrome coronavirus 2, M: male, F: female, PMH: past medical history, HTN: hypertension, DM: diabetes mellitus, ICU: intensive care unit, NA: not applicable.

† Psychiatric disorders, including depressive disorders, anxiety disorders, trauma- and stress-related disorders.

‡ Cardiovascular disorders except for HTN

§ The effect of the vaccine platform on post-SARS-CoV-2 headaches is reported only for headaches following the same vaccine dose.

‖ The effect of using analgesics before vaccination on post-SARS-CoV-2 headaches is reported only for headaches following the same vaccine dose.

¶ The effect of post-vaccination fever on post-SARS-CoV-2 headaches is reported only for headaches following the same vaccine dose.

* Significant at P < 0.05

** Significant at P < 0.001

*** Significant at P < 0.001

Table S8. Unadjusted impact of variables on developing prolonged headache following SARS-CoV-2 vaccination

Variables	1st dose		2nd dose		3rd dose	
	OR (95% CI)	P	OR (95% CI)	P	OR (95% CI)	P
**Age**	1.05 (1.03, 1.07)	**<0.001*** **	1.01 (0.98, 1.04)	0.538	0.98 (0.92, 1.03)	0.423
**Sex (F vs. M)**	2.13 (1.33, 3.45)	**0.002** **	1.23 (0.67, 2.33)	0.500	1.33 (0.36, 5.00)	0.661
**PMH**						
Thyroid disorders	0.62 (0.24, 1.39)	0.273	1.02 (0.29, 3.73)	0.975	NA	NA
Psychiatric disorders†	2.65 (1.12, 6.41)	**0.027***	0.85 (0.10, 7.29)	0.873	0.92 (0.10, 8.71)	0.941
HTN	1.58 (0.67, 3.61)	0.285	1.13 (0.24, 5.98)	0.874	NA	NA
Autoimmune disorders	1.32 (0.44, 3.64)	0.594	2.55 (0.32, 52.42)	0.424	1.85 (0.16, 42.79)	0.634
Respiratory disorders	0.80 (0.22, 2.39)	0.710	0.51 (0.10, 2.18)	0.372	0.92 (0.03, 25.12)	0.957
Cardiovascular disorders‡	2.94 (0.64, 15.10)	0.161	NA	NA	NA	NA
DM	1.10 (0.15, 5.72)	0.910	0.42 (0.02, 4.56)	0.490	NA	NA
**Primary headaches history**	0.64 (0.46, 0.90)	**0.010***	1.36 (0.80, 2.32)	0.254	1.66 (0.47, 6.10)	0.434
**Primary headaches type**						
Migraine-like vs. Tension-type	0.84 (0.52, 1.37)	0.488	0.86 (0.42, 1.74)	0.676	0.50 (0.08, 2.75)	0.434
Cluster vs. Tension-type	NA	NA	0.73 (0.19, 2.86)	0.644	NA	NA
**History of COVID-19 infection**	0.86 (0.62, 1.21)	0.392	1.01 (0.60, 1.69)	0.981	1.05 (0.28, 3.96)	0.936
**COVID-19 severity**						
Ward admission vs. home quarantine	1.03 (0.35, 2.70)	0.956	2.00 (0.37, 14.95)	0.437	NA	NA
ICU admission vs. home quarantine	NA	NA	NA	NA	NA	NA
**COVID-19 manifestation**						
Systemic vs. Respiratory	1.15 (0.62, 2.12)	0.647	1.67 (0.66, 4.29)	0.281	1.04 (0.17, 6.77)	0.968
Gastrointestinal vs. Respiratory	1.48 (0.74, 2.92)	0.256	0.75 (0.21, 2.51)	0.642	0.26 (0.01, 2.54)	0.284
Neurological vs. Respiratory	1.00 (0.39, 2.40)	1.000	0.40 (0.05, 2.08)	0.303	NA	NA
**History of COVID-19 headaches**	0.01 (0.00, 0.04)	**<0.001*** **	1.32 (0.59, 2.97)	0.499	1.33 (0.25, 7.12)	0.730
**COVID-19 headaches type**						
Migraine-like vs. Tension-type	NA	NA	1.48 (0.45, 4.94)	0.518	1.29 (0.13, 13.07)	0.822
Undifferentiated vs. Tension-type	NA	NA	0.18 (0.01, 1.42)	0.150	NA	NA
**COVID-19 headaches intensity**						
Moderate vs. Mild	NA	NA	NA	NA	0.80 (0.03, 24.81)	0.887
Severe vs. Mild	NA	NA	NA	NA	2.33 (0.07, 74.49)	0.590
**COVID-19 headaches time to onset**						
<1 d vs. Manifesting	NA	NA	0.90 (0.18, 4.21)	0.892	1.20 (0.13, 12.83)	0.872
1≤onset<2 d vs. Manifesting	NA	NA	0.33 (0.05, 1.78)	0.207	4.50 (0.30, 137.89)	0.307
2≤onset<7 d vs. Manifesting	NA	NA	0.57 (0.10, 3.00)	0.511	NA	0.996
≥7 d vs. Manifesting	NA	NA	NA	NA	NA	NA

**COVID-19 headaches attack duration**	0.24 (0.00, 0.82)	0.422	0.99 (0.96, 1.03)	0.602	1.01 (0.96, 1.09)	0.651
**COVID-19 headaches attack duration, categorical**						
1≤duration<4 h vs. <1 h	NA	NA	NA	0.994	NA	NA
4≤duration<24 h vs. <1 h	NA	NA	NA	0.995	NA	NA
24≤duration<72 h vs. <1 h	NA	NA	NA	0.994	NA	NA
≥72 h vs. <1 h hour	NA	NA	NA	0.995	NA	NA
**COVID-19 headaches end**	NA	NA	1.02 (0.98, 1.10)	0.380	0.97 (0.83, 1.11)	0.642
**COVID-19 headache end, categorical**						
≥48 h vs. <48 h	NA	NA	NA	NA	NA	NA
**History of post-1st dose headaches**	-	-	1.31 (0.79, 2.19)	0.296	0.71 (0.21, 2.45)	0.592
**Post-1st dose headaches type**						
Migraine-like vs. Tension-type	0.69 (0.47, 1.00)	0.050	1.62 (0.79, 3.35)	0.186	0.42 (0.04, 3.13)	0.407
Undifferentiated vs. Tension-type	0.71 (0.44, 1.14)	0.164	1.57 (0.44, 5.90)	0.487	NA	0.994
**Post-1st dose headaches intensity**						
Moderate vs. Mild	-	-	1.35 (0.39, 4.67)	0.627	3.33 (0.22, 92.98)	0.398
Severe vs. Mild	-	-	1.57 (0.45, 5.56)	0.476	2.00 (0.12, 58.79)	0.638
**Post-1st dose headaches intensity**						
Severe vs. Non-severe	-	-	1.26 (0.53, 3.00)	0.601	0.83 (0.11, 6.40)	0.858
**Post-1st dose headaches time to onset**						
1≤onset<2 d vs. <1 d	-	-	1.71 (0.64, 4.92)	0.294	NA	NA
2≤onset<7 d vs. <1 d	-	-	3.00 (0.37, 61.71)	0.348	NA	NA
≥7 d vs. <1 d	-	-	4.00 (0.57, 79.64)	0.222	NA	NA
**Post-1st dose headaches attack duration**	-	-	0.99 (0.91, 1.05)	0.663	1.01 (0.87, 1.16)	0.904
**Post-1st dose headaches attack duration,**						
1≤duration<4 h vs. <1 h	-	-	1.89 (0.17, 41.71)	0.611	0.33 (0.01, 4.66)	0.437
4≤duration<24 h vs. <1 h	-	-	3.00 (0.26, 68.26)	0.388	1.00 (0.03, 34.43)	1.000
24≤duration<72 h vs. <1 h	-	-	1.00 (0.02, 40.40)	1.000	NA	NA
≥72 h vs. <1 h hour	-	-	6.00 (0.38, 182.92)	0.224	0.33 (0.01, 4.66)	0.437
**Post-1st dose headaches end**	-	-	1.01 (1.00, 1.02)	0.127	1.92 (1.07, 7.60)	0.158
**Post-1st dose headaches end, categorical**						
≥48 h vs. <48 h	-	-	3.20 (1.16, 10.33)	**0.033***	NA	NA
**History of post-2nd dose headaches**	-	-	-	-	1.10 (0.31, 3.95)	0.879
**Post-2nd dose headaches type**						
Migraine-like vs. Tension-type	-	-	1.82 (1.05, 3.19)	**0.035***	0.94 (0.12, 7.07)	0.949
Undifferentiated vs. Tension-type	-	-	0.92 (0.42, 1.99)	0.840	NA	NA
**Post-2nd dose headaches intensity**						
Moderate vs. Mild	-	-	-	-	0.38 (0.01, 5.92)	0.497
Severe vs. Mild	-	-	-	-	0.75 (0.02, 15.10)	0.851

**Post-2nd dose headaches intensity**						
Severe vs. Non-severe	-	-	-	-	1.50 (0.17, 15.56)	0.715
**Post-2nd dose headaches time to onset**						
1≤onset<2 d vs. <1 d	-	-	-	-	NA	NA
2≤onset<7 d vs. <1 d	-	-	-	-	NA	NA
≥7 d vs. <1 d	-	-	-	-	NA	NA
**Post-2nd dose headaches attack duration**						
1≤duration<4 h vs. <1 h	-	-	-	-	NA	NA
4≤duration<24 h vs. <1 h	-	-	-	-	0.33 (0.01, 4.66)	0.437
24≤duration<72 h vs. <1 h	-	-	-	-	NA	NA
≥72 h vs. <1 h hour	-	-	-	-	NA	NA
**Post-2nd dose headaches end**	-	-	-	-	1.08 (1.01, 1.22)	0.097
**Post-2nd dose headaches end, categorical**						
≥48 h vs. <48 h	-	-	-	-	6.00 (0.56, 145.57)	0.170
**Post-3rd dose headaches type**						
Migraine-like vs. Tension-type	-	-	-	-	5.07 (1.18, 27.32)	**0.038***
Undifferentiated vs. Tension-type	-	-	-	-	2.67 (0.09, 84.49)	0.532
**Vaccine platform**[§]						
Vector vs. Inactivated	2.64 (1.71, 4.19)	**<0.001*** **	0.60 (0.34, 1.05)	0.075	0.53 (0.07, 3.16)	0.503
Protein Subunit vs. Inactivated	NA	NA	NA	NA	0.50 (0.03, 6.96)	0.600
mRNA vs. Inactivated	0.48 (0.03, 2.70)	0.493	0.38 (0.07, 1.68)	0.211	NA	NA
**Analgesics use before vaccination**[‖]	2.20 (1.41, 3.45)	**<0.001*** **	1.72 (0.89, 3.39)	0.111	0.24 (0.01, 2.09)	0.238
**Post-vaccination fever**[¶]	0.96 (0.68, 1.35)	0.799	0.90 (0.54, 1.50)	0.694	0.61 (0.15, 2.51)	0.493

Note: Abbreviations: OR: odds ratio, CI: confidence interval, P: probability value, COVID-19: coronavirus disease 2019, SARS-CoV-2: severe acute respiratory syndrome coronavirus 2, M: male, F: female, PMH: past medical history, HTN: hypertension, DM: diabetes mellitus, ICU: intensive care unit, NA: not applicable

† Psychiatric disorders, including depressive disorders, anxiety disorders, trauma- and stress-related disorders.

‡ Cardiovascular disorders except for HTN

§ The effect of the vaccine platform on post-SARS-CoV-2 headaches is reported only for headaches following the same vaccine dose.

|| The effect of using analgesics before vaccination on post-SARS-CoV-2 headaches is reported only for headaches following the same vaccine dose.

¶ The effect of post-vaccination fever on post-SARS-CoV-2 headaches is reported only for headaches following the same vaccine dose.

* Significant at P < 0.05

** Significant at P < 0.001

*** Significant at P < 0.001
